# Osteogenic transdifferentiation of primary human fibroblasts to osteoblast-like cells with human platelet lysate

**DOI:** 10.1038/s41598-022-18512-1

**Published:** 2022-08-29

**Authors:** Ferdy K. Cayami, Lauria Claeys, Ruben de Ruiter, Bernard J. Smilde, Lisanne Wisse, Natalija Bogunovic, Elise Riesebos, Lyra Eken, Irsan Kooi, Erik A. Sistermans, Nathalie Bravenboer, Gerard Pals, Sultana M. H. Faradz, Daoud Sie, E. Marelise W. Eekhoff, Dimitra Micha

**Affiliations:** 1 Department of Human Genetics, Amsterdam UMC, Amsterdam Movement Sciences, Vrije Universiteit Amsterdam, Amsterdam, The Netherlands; 2 Center for Biomedical Research (CEBIOR), Faculty of Medicine, Diponegoro University, Jln Prof Soedarto SH, 50275 Semarang, Indonesia; 3 Department of Internal Medicine, Section Endocrinology, Amsterdam UMC, Amsterdam Movement Sciences, Vrije Universiteit Amsterdam, Amsterdam, The Netherlands; 4 Department of Clinical Chemistry, Amsterdam UMC, Amsterdam Movement Sciences, Vrije Universiteit Amsterdam, Amsterdam, The Netherlands

**Keywords:** Mechanisms of disease, Transdifferentiation

## Abstract

Inherited bone disorders account for about 10% of documented Mendelian disorders and are associated with high financial burden. Their study requires osteoblasts which play a critical role in regulating the development and maintenance of bone tissue. However, bone tissue is not always available from patients. We developed a highly efficient platelet lysate-based approach to directly transdifferentiate skin-derived human fibroblasts to osteoblast-like cells. We extensively characterized our in vitro model by examining the expression of osteoblast-specific markers during the transdifferentiation process both at the mRNA and protein level. The transdifferentiated osteoblast-like cells showed significantly increased expression of a panel of osteogenic markers. Mineral deposition and ALP activity were also shown, confirming their osteogenic properties. RNA-seq analysis allowed the global study of changes in the transcriptome of the transdifferentiated cells. The transdifferentiated cells clustered separately from the primary fibroblasts with regard to the significantly upregulated genes indicating a distinct transcriptome profile; transdifferentiated osteoblasts also showed significant enrichment in gene expression related to skeletal development and bone mineralization. Our presented in vitro model may potentially contribute to the prospect of studying osteoblast-dependent disorders in patient-derived cells.

## Introduction

Bone disorders encompass a spectrum of etiology which extends from inherited bone disorders such as skeletal dysplasia, with an established monogenic cause, to disorders with a less defined genetic contribution such as the highly prevalent postmenopausal osteoporosis^1,2^. In order to build knowledge on the underlying disease mechanism and identify points for therapeutic intervention, relevant cell models are necessary in which the pathological condition is reflected and can be studied. The acquisition of bone tissue from affected individuals is not always possible so suitable alternatives are being sought for diagnostic and research purposes. The development of a method to noninvasively produce bone cells from skin fibroblasts of a larger group of human donors may help to address this need.

Bone homeostasis is based on bone remodeling which includes formation and development throughout life in combination with adaptation to mechanical demands^3^. Bone tissue is maintained by osteoblasts, osteoclasts and osteocytes^4^. Osteoblasts are responsible for the formation of bone tissue through the deposition of bone extracellular matrix which becomes mineralized by calcium hydroxyapatite to confer bone its strength and rigidity^5^. Osteoclasts serve an opposing role by mediating bone resorption. Osteoblasts differentiate terminally to osteocytes which respond to mechanical stimuli by activating osteoblasts or osteoclasts to induce an anabolic or catabolic effect respectively. The function of these three types of cells is crucial in preserving the bone structure and providing mechanical resilience against environmental factors; this largely depends on the amount and consistency of the extracellular matrix produced by the osteoblasts^4^. Given the critical contribution of osteoblasts to bone formation and their role as osteocyte precursors, molecular insight into these cells in a relevant disease model is essential in order to comprehend bone diseases.

The study of bone diseases, notably hereditary bone disorders, is severely hindered by the scarcity of human bone tissue. Acquirement of bone tissue requires a skilled operator and is an invasive procedure with risks and inconvenience for the patients. In some rare bone diseases such as Fibrodysplasia Ossificans Progressiva (FOP), biopsy can result in detrimental complications, as the tissue injury can induce heterotopic ossification after the biopsy^6^. On the other hand, the acquisition of skin biopsies for the establishment of dermal fibroblast cultures is relatively easy and has a lower risk of complications.

Recent developments in the field of induced pluripotent stem cells (iPSCs) enables dedifferentiation of fibroblasts back to stem cells which can be further differentiated to the intended cell type including osteoblasts^7^. Although several studies have been performed demonstrating the differentiation of osteoblasts from iPSCs, the process remains technically laborious and time-consuming^8–10^. Expression of the reprogramming factors for iPSC generation by means of viral transduction can modify the genetic properties of cells which is undesirable for the study of several genetic diseases^9,11^. Studies to produce osteoblast-like cells from fibroblasts also involve the use of growth factors and small molecules^12–14^. Another approach to study osteoblasts is through differentiation from their lineage cells, the mesenchymal stem cells (MSCs), which are commonly obtained from bone marrow. However, even though the isolation, processing and culturing of MSCs is possible, MSCs pose the challenge of losing their differentiation potency and presenting the probability of malignant transformation at higher passages^15^. In addition to bone marrow, MSCs are also obtainable from adipose tissue and umbilical cord blood-derived stem cells (UCBSCs) which cannot be applied to all donors^16^. Given the limitations of these techniques, we exploited the MSC properties of fibroblasts by transdifferentiating human primary skin fibroblasts directly to osteoblast-like cells, thereby bypassing the requirement of the intermediate stem cell state^17,18^. Since platelet lysate is known to promote the osteogenic differentiation of MSCs, we hypothesized that it can be applied to the osteogenic transdifferentiation of dermal fibroblasts which share MSC characteristics^19^. In this study, we characterize the production of osteoblast-like cells from primary dermal fibroblasts. This study provides detailed characterization of the differentiated cells and the differentiation process which is important for future applications of these cells as a disease model. Moreover, this study delivers new insights on the utility of human platelet lysate for the direct differentiation of fibroblasts towards osteoblast-like cells by osteogenic transdifferentiation.

## Results

### Characterization of the transdifferentiated cells

In order to fully characterize the nature and efficiency of the osteogenic transdifferentiation, the converted osteoblast-like cells were interrogated for the expression of a panel of osteogenic markers which are known to be uniquely expressed in osteoblasts at different stages of their differentiation from MSCs. In addition, their functional resemblance to osteoblasts was also confirmed by testing of in vitro mineralization.

### In vitro mineralization

**Figure 1:**
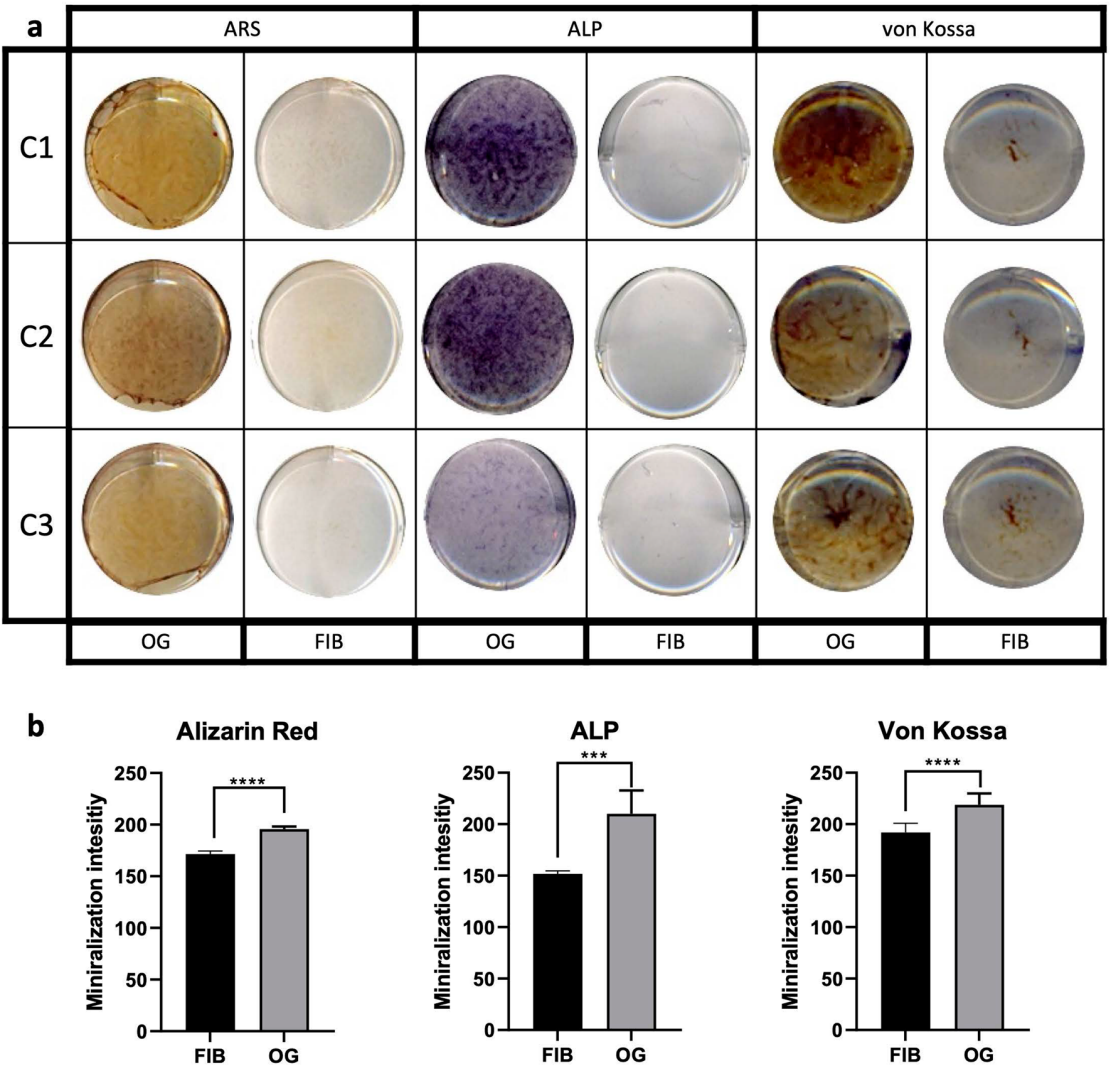

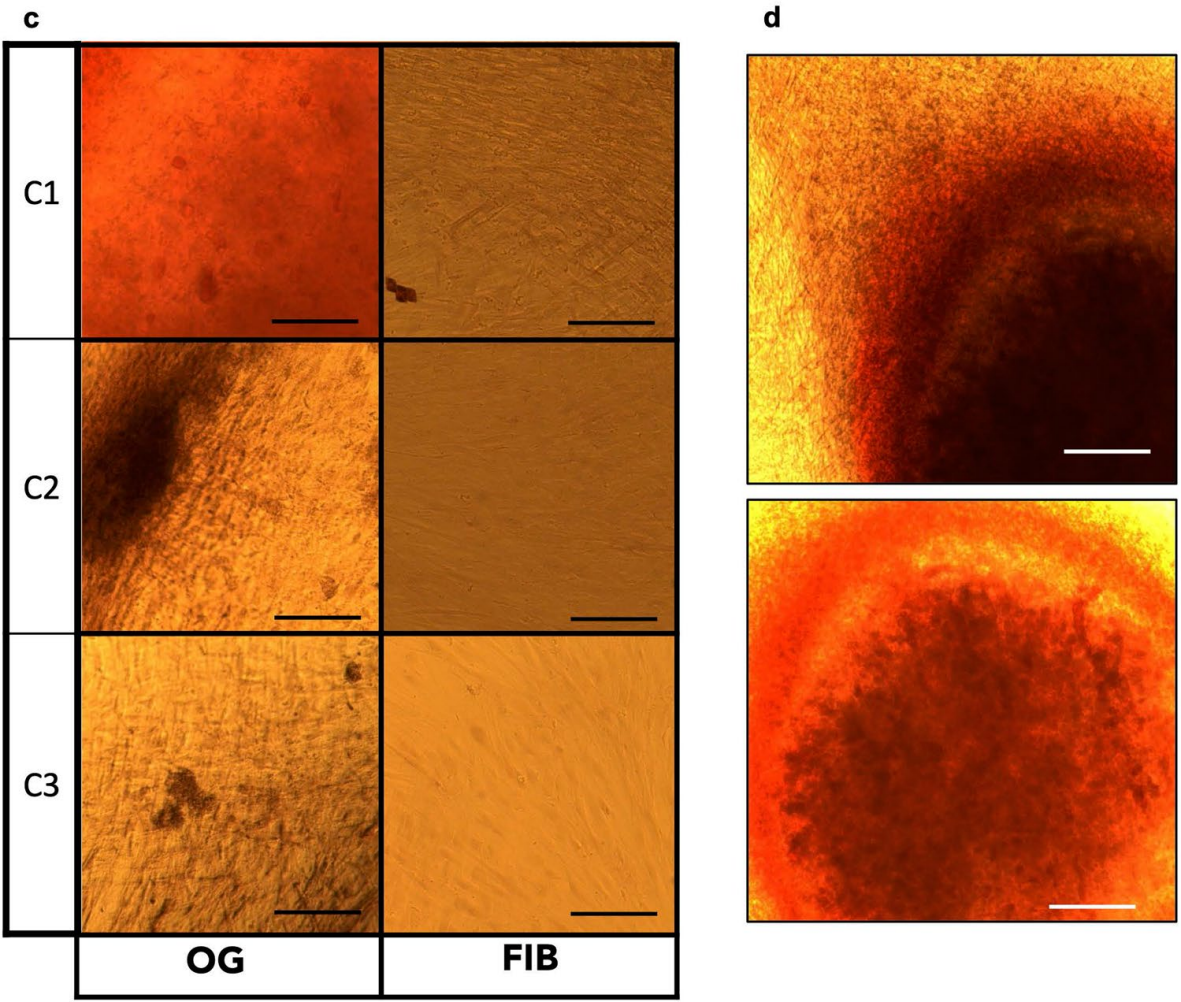
Functional staining for Alizarin red (ARS), Alkaline phosphatase (ALP) activity and von Kossa to show mineralization properties of transdifferentiated osteoblast-like cells from healthy donors. Primary fibroblasts from healthy donors were subjected to osteogenic transdifferentiation (OG) for 21 days; undifferentiated fibroblasts (FIB) were grown in fibroblast medium for 21 days. (**a**) Photos show representative results for 3 primary fibroblast cell cultures (C1, C2, C3). ARS and von Kossa stainings (brown) indicate calcium phosphate deposition while positive ALP staining staining (purple) shows ALP activity required for mineralization. (**b**) The intensity of the staining was quantified for ARS in 6 different primary fibroblast cell culturess, ALP and von Kossa in 9 different primary fibroblast cell cultures. Bars indicate the mean of the staining and error bars the standard deviation. Asterisks *** indicate *p* < 0.005 as measured by ANOVA. (**c**) Microscopic images of 3 fibroblast controls (C1, C2, C3) in the different media after 21 days of culture. (**d**) Magnified images of the mineralized nodules of osteoblast-like cells shown by Alizarin red staining. Black scale bar represents 200 µm while white scale bar represents 50 µm.

To determine the transdifferentiation of fibroblasts to osteoblasts, we demonstrated the mineralization capability of the transdifferentiated osteoblast-like cells on a functional level by alizarin red S (ARS) and von Kossa staining which indicate calcium phosphate deposition. Alkaline phosphatase (ALP) activity assay was also performed in which mineralization was related to activation of ALP, a known mediator of mineral deposition^20^. As depicted in Fig. 1a, the osteoblast-like cells from healthy skin biopsy donors showed positive staining for all three functional tests. Although there was some variability in the intensity of the staining, all control cells showed positive staining in response to 21 days treatment with the osteogenic medium; this was absent in cells in the fibroblast medium. The presence of mineralization and ALP activity after osteogenic transdifferentiation was also confirmed by quantification of the staining which was shown to be significant (Fig. 1b). Microscopy images of the ARS assay detected the formation of mineralized nodules as illustrated in Fig. 1c and d, also a characteristic property of cells with osteogenic properties. In addition, the effect of the osteogenic media on induction of mineralization was also tested in primary human osteoblasts; a similar level of mineralization was observed in relation to the transdifferentiated osteoblast-like cells (Supplemental Fig. 1).

### Protein expression of osteogenic markers

**Figure 2:**
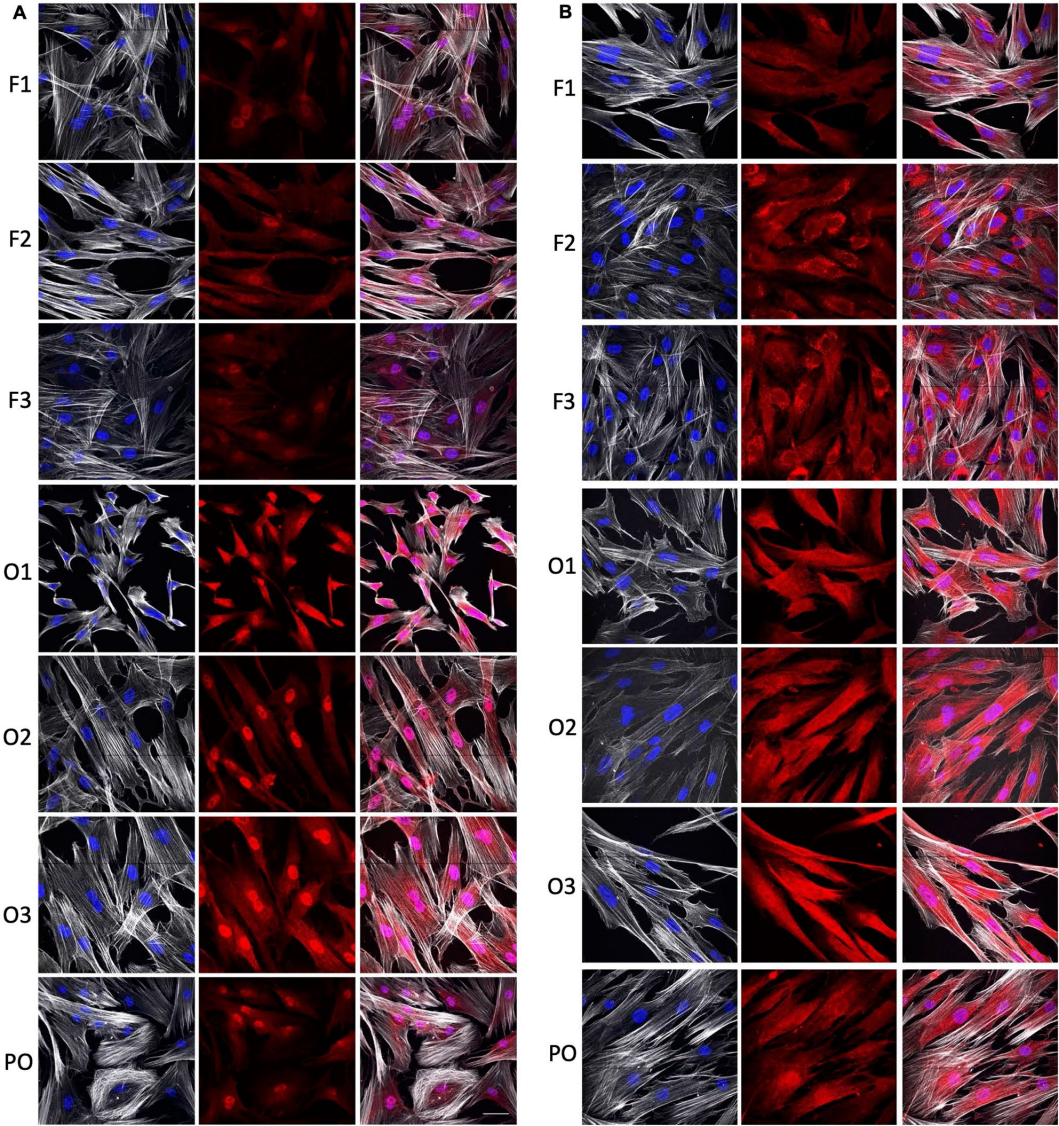
Immunofluorescence staining of RUNX2 and osteocalcin in transdifferentiated osteoblast-like cells. Representative images of 3 primary cell cultures from healthy donors before (F1, F2, F3) and after 21 days of osteogenic transdifferentiation (O1, O2, O3); PO indicates human primary osteoblasts. (**A**) Immunofluorescence staining of RUNX2 in fibroblasts and transdifferentiated osteoblast-like cells. The first column shows DAPI (blue) and phalloidin (white), the second column shows RUNX2 (red), the third column is the merged image of the described two columns and the fourth column is the merged image with a lower magnification. Scale bar is representative of 50 µm. (**B**) Immunofluorescence staining of osteocalcin in fibroblasts and transdifferentiated osteoblast-like cells. The first column shows DAPI (blue) and phalloidin (white), the second column shows osteocalcin (red), the third column is the merged image of the described two columns.

**Figure 3:**
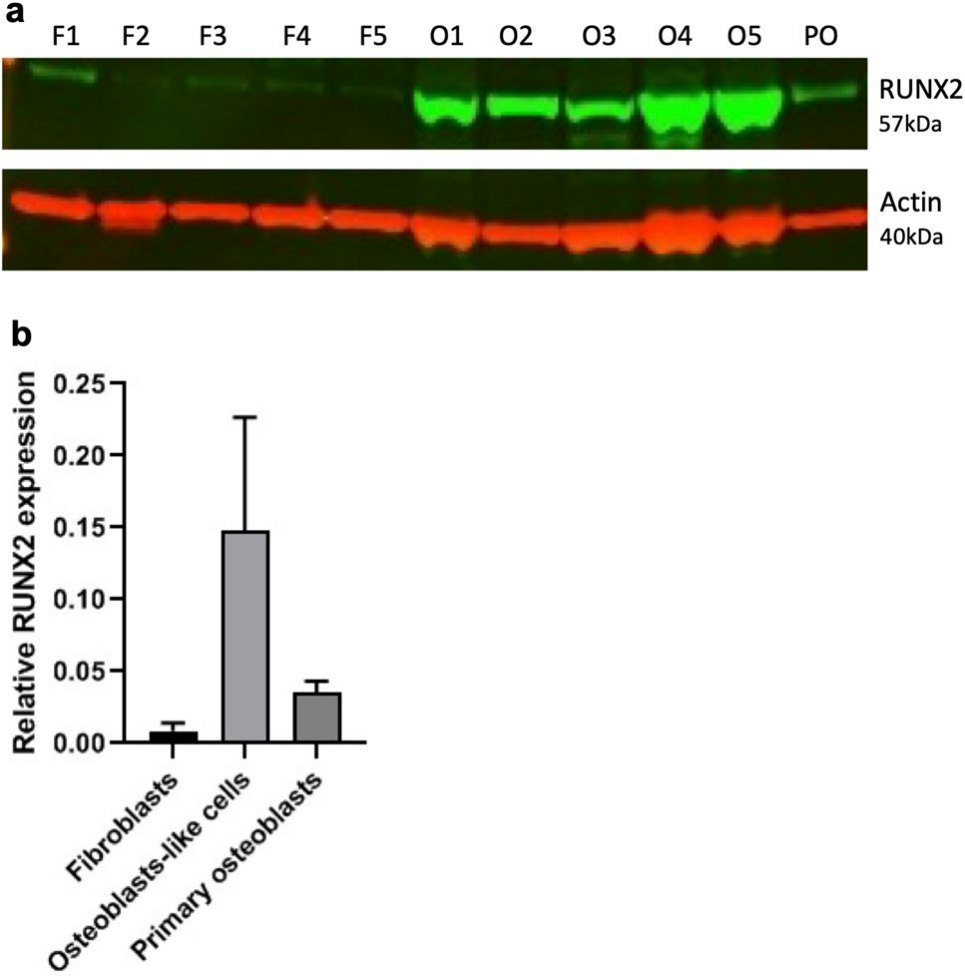
Western blot analysis of RUNX2 expression. (**a**) RUNX2 expression determined by western blotting analysis of fibroblasts from 5 healthy controls (F1-F5) and their transdifferentiated osteoblast-like cells on day 21 of osteogenic transdifferentiation (O1-O5) and primary osteoblasts (PO). Actin was used a loading control. (**b**) The western blot was also quantified. The full-length blot can be seen in supplemental Fig. 10.

To further characterize the osteoblast-like cells, immunostaining was carried out to detect expression of osteoblast markers in primary fibroblasts of 6 healthy individuals subjected to osteogenic transdifferentiation for 21 days. Runt-related transcription factor 2 (RUNX2) is the first transcription factor to determine commitment to the osteoblast cell lineage and trigger the expression of other major bone matrix genes. It is known to be expressed higher in earlier stages of osteoblast differentiation and then lowers in mature osteoblasts. The expression of *RUNX2* was higher in osteoblast-like cells while it was barely detectable in the primary fibroblasts (Fig. 2A, Supplemental Fig. 2). The second investigated marker is Osteocalcin (OC), encoded by *BGLAP* gene; this is a bone-specific protein uniquely secreted by mature osteoblasts and osteocytes. It is also the most abundant non-collagenous protein found in bone. OC was found to be upregulated in the osteoblast-like cells (Fig. 2B, Supplemental Fig. 2). This indicates that the osteogenic transdifferentiation generated osteoblast-like cells which display expression of both OC and RUNX2. As expected, OC was mostly expressed in the cytoplasm while RUNX2 in the nucleus. In particular, the upregulation in the expression of RUNX2 in the transdifferentiated osteoblast-like cells was also confirmed by western blotting analysis compared to the primary fibroblasts (Fig. 3). Regarding the shape morphology of the cells, no significant changes were found after quantification (Supplemental Fig. 3). To determine the efficiency of the transdifferentiation, we quantified the RUNX2 and OC positively stained cells in the osteogenic medium. The average percentage of positive cells after osteogenic transdifferentiation was 86 ± 6.1% and 88 ± 8.7% for RUNX2 and OC respectively (Supplemental Fig. 4).

### Expression of osteogenic markers on mRNA level

**Figure 4:**
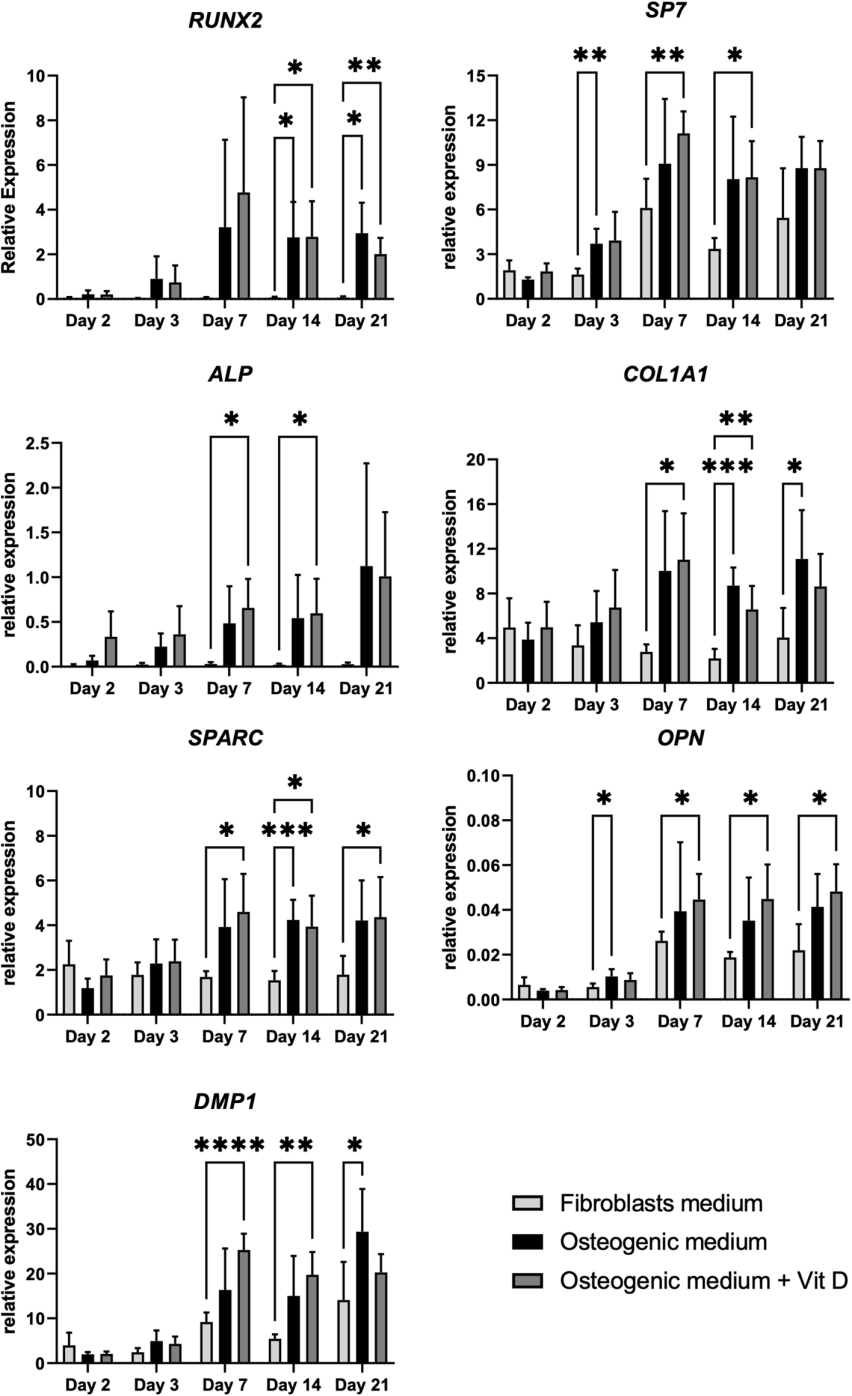
Osteogenic marker mRNA expression in healthy primary cell cultures during transdifferentiation to osteoblast-like cells. Primary fibroblasts from 6 healthy donors were cultured in fibroblast medium or osteogenic medium with and without vitamin D for 21 days during which osteoblast marker expression was quantified on days 2, 3, 7, 14 and 21. The expression of osteoblast marker genes *RUNX2*, *SP7*, *ALP*, *COL1A1*, *SPARC*, *OPN*, and *DMP1* was measured by qPCR and normalized to the housekeeping gene *YWHAZ*. Values are expressed as mean ± SEM (n = 6 per medium condition). Asterisks * indicate statistically significant differences between the indicated conditions (*p* ≤ 0.05).

The next question we addressed was the expression of the osteogenic markers on mRNA level. The expression of bone-specific genes which are known to be expressed at different time points during the differentiation of MSCs to osteoblasts was measured by quantitative PCR (qPCR). Given that vitamin D and its metabolites are known to stimulate bone formation, we also investigated if vitamin D can boost the osteogenic transdifferentiation process^21^. After differentiation in osteogenic media, osteoblast-like cells showed significantly higher mRNA expression of all osteogenic markers (*RUNX2*, *SP7* (Osterix), *SPARC* (Osteonectin), *ALP*, *OPN*, *COL1A1*, *DMP1*) compared to undifferentiated primary fibroblasts starting from day 3. As depicted in Fig. 4, addition of vitamin D to the osteogenic medium did not have a significant effect on the osteogenic transdifferentiation of cells as shown by the expression of the osteogenic markers compared to osteogenic medium alone. For all experiments in this study osteogenic transdifferentiation was conducted with 5% platelet lysate which has been shown to optimally induce the transdifferentiation process (Supplemental Fig. 5).

**Figure 5:**
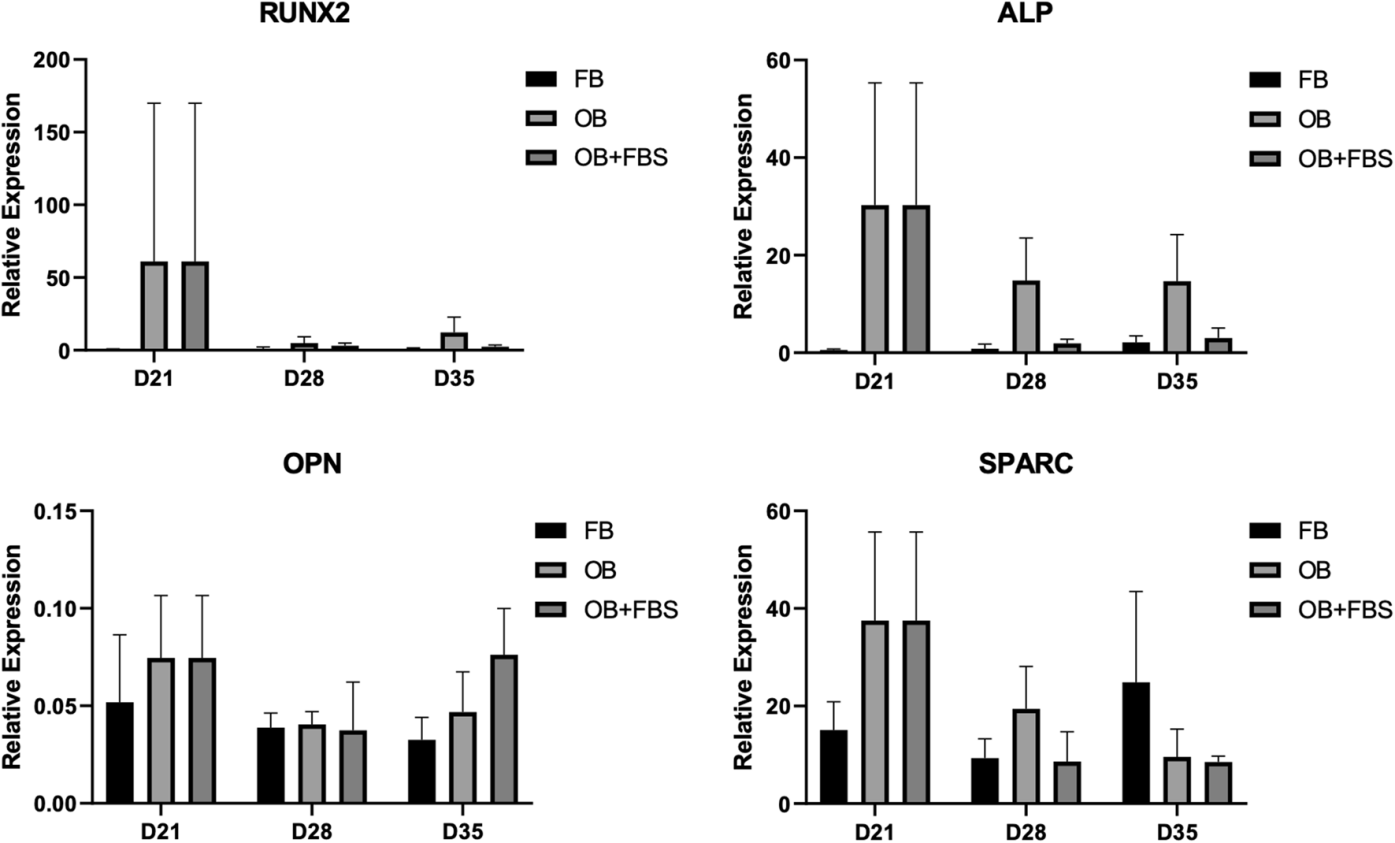
Level of maintenance of osteogenic transdifferentiation per primary cell cultures after day 21. The expression of *RUNX2*, *ALP*, *SPARC* and *OPN* was measured in 5 different primary cell cultures from healthy donors on day 28 and 35 after osteogenic transdifferentiation for 21 days, after which the cells were further maintained in the osteogenic media with 5% platelet lysate (OG) or osteogenic media with 10% FBS (FBS); at the indicated timepoints cells were also harvested after culture in fibroblast media (FIB). The expression was measured by qPCR and results were normalized based on the expression of *TBP*. Error bars indicate the SEM per group of cells.

After the 21-day osteogenic transdifferentiation period, the maintenance of the differentiated state differed for each primary cell culture until day 35 (Fig. 5). Most primary fibroblast cell cultures with supplementation of the osteogenic media with platelet lysate showed higher expression of ALP on day 28 and 35 compared to the untreated fibroblasts or cells treated with osteogenic media and FBS. A similar pattern was noted for the expression of *RUNX2* on day 35 in all primary cell cultures which was increased in osteogenic media with platelet lysate compared to osteogenic media with FBS whereas no consistent differences were noted for the expression of *SPARC* and *OPN* (Fig. 5). The transdifferentiated osteoblast-like cells on day 21 were also compared to primary human osteoblasts. The latter had lower expression of *RUNX2, ALP, SPARC, SP7, COL1A1 and DMP1* but increased expression of the late osteoblast marker OPN (Supplemental Fig. 6). Gene expression of *RUNX2* and *ALP* was also investigated in MSCs after treating them with the platelet lysate-based osteogenic media. Results showed that the osteogenic media induced osteoblast differentiation as seen by the increased expression of *RUNX2* and *ALP* compared to the untreated MSCs (Supplemental Fig. 7).

### Global mRNA analysis by RNA-seq

**Figure 6:**
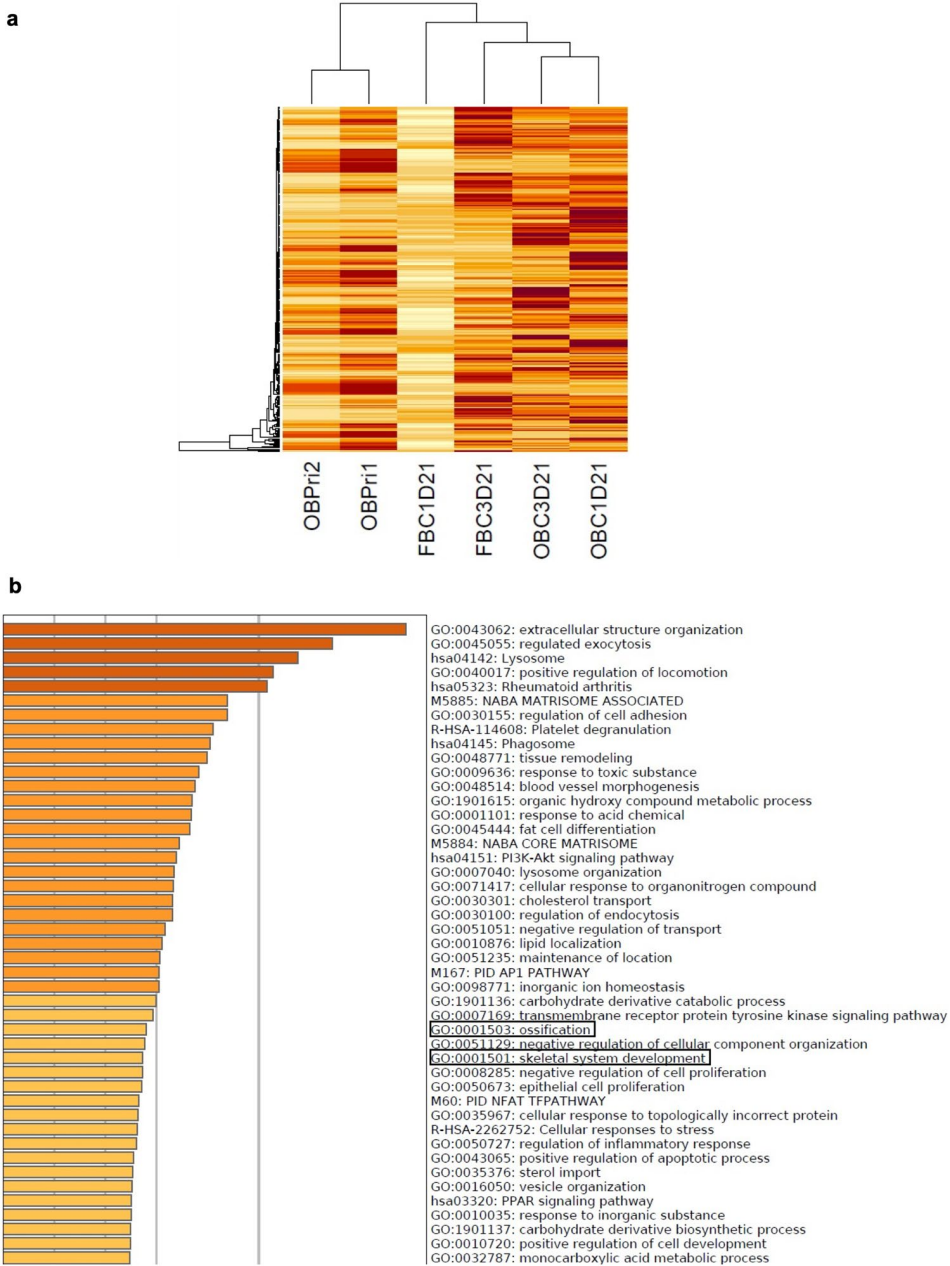
RNA-seq was used to investigate mRNA expression on day 21 in osteogenic media and fibroblast media. Profile of global mRNA expression during osteogenic transdifferentiation. Cells from 2 healthy controls (FIB) were cultured in osteogenic medium (OG) and fibroblast medium (Fib) for 21 days. (**a**) Heatmap shows an overview of significantly upregulated genes in osteogenic medium compared to fibroblast medium indicating separate clustering of cells in fibroblast medium on day 21 (middle 2 columns) compared to cells in osteogenic medium on day 21 (right 2 columns) and primary osteoblasts (left 2 columns). (**b**) Gene ontology results show significant changes in upregulation of gene expression related to bone function.

To gain more information on the global mRNA expression profile of the osteoblast-like cells and perchance recognize the pathways which are activated by platelet lysate during osteogenic transdifferentiation, RNA sequencing (RNA-seq) was performed on healthy primary fibroblast cells, osteoblast-like cells after 21 days of transdifferentiation and primary osteoblasts. Based on the significantly upregulated genes in the osteoblast-like cells and primary osteoblasts, primary fibroblast cells clustered together as depicted in Fig. 6. In total 16,266 genes were detected in the RNA-seq. 358 genes were significantly different between primary fibroblast cells and osteoblast-like cells and 1,514 genes were significantly different between primary fibroblast cells and primary osteoblasts. Approximately half of the genes differentially expressed in osteoblast-like cells compared to primary fibroblasts (198) were also differentially expressed in primary osteoblasts, which is a significant enrichment (*p* < 0.0001) (Supplemental Table 1; Supplemental Fig. 8). Moreover, both osteoblast-like cells and osteoblasts show a significant enrichment of genes linked to osteoblasts based on comparison to a harmonizome data set which used text mining to identify 833 genes associated with osteoblasts (*p* < 0.0001). Metascape gene ontology analysis showed in the significantly regulated genes of osteoblast-like cells and primary osteoblasts compared to primary fibroblasts an enrichment for bone related pathways such as skeletal system development and ossification (Fig. 6). Our RNA-seq data confirmed upregulation of 5 genes involved in osteoblast differentiation such as *ALP*, *COL1A1*, *SERPINF1*, *CRTAP* and *P3H1* after transdifferentiation of the primary fibroblasts to osteoblast-like cells (*p* > 0.05). Moreover, the data showed higher expression of *RUNX2*, *COL1A1* and *CRTAP* in primary osteoblasts compared to primary fibroblasts (*RUNX2* *p* < 0.05). No significantly higher expression of the chondrogenic markers *SOX9*, *ACAN* and *COL2A1* was found in the transdifferentiated osteoblasts compared to primary fibroblasts by RNA-seq analysis. Also, no differences were found in the expression of *SOX9* and *ACAN* between primary fibroblasts and osteoblast-like cells by qPCR analysis (Supplemental Fig. 9)^22^.

## Discussion

Different strategies have been proposed to differentiate fibroblasts to cells of osteogenic cell lineage including transgene-mediated and transgene-free methods^23–26^. In the present study, we transdifferentiated skin-derived primary human fibroblasts directly to osteoblast-like cells which was confirmed by the presence of mineralization, expression of bone-relevant markers and morphologically. To avoid the pitfalls of von Kossa staining in visualizing mineralization, we performed additional stainings such as ARS and ALP activity to demonstrate the osteogenic properties of our osteoblast-like cells. Both ARS and von Kossa staining clearly displayed the ability of the osteoblast-like cells to deposit calcium phosphate, a fundamental property of osteoblasts^27,28^. Considering that ALP is an important enzyme for osteoblasts to initiate calcification and promote mineralization, positive staining for ALP activity further confirmed the osteogenic properties of the transdifferentiated osteoblast-like cells^5,20^.

RUNX2 is known as the main transcription factor which induces differentiation of MSCs to osteoblasts^29–33^. As previously described, RUNX2 is localized in the nucleus to physiologically promote differentiation to osteoblasts^34^. As expected, immunofluorescence analysis of osteoblast-like cells showed RUNX2 localization in the nucleus whereas the staining was negative in the undifferentiated primary fibroblast cells. Interestingly, some transdifferentiated cells also showed slight positive staining of RUNX2 in the cytoplasm which has been previously reported in osteocytes of woven bone, marrow stromal cells and periosteum cells from bone biopsies^35^. Whether this indicates differentiation to other types of bone-related cells still remains to be investigated. Furthermore, the formation of osteoblast-like cells was also supported by the detection of OC expression. OC is the most abundant non-collagen protein in the bone known to be essential in bone turnover and mineralization^36–38^. As previously reported, transcription of OC is regulated through dependency to RUNX2; the concomitant expression of both was shown in our transdifferentiated osteoblast-like cells^39^.

We measured mRNA expression of the reported osteoblast markers to further determine the specificity of our osteoblast-like cells. In general, the osteoblast-like cells significantly expressed all the osteogenic markers (*RUNX2*, *SP7*, *SPARC*, *ALP*, *OPN*, *COL1A1*, *DMP1*) in comparison to the primary fibroblasts after 21 days in culture as depicted in Fig. 3. The addition of vitamin D did not result in enhanced osteogenic transdifferentiation for any of these markers so its use is not required in this method. The combined expression of *RUNX2* and *SP7* is shown to be crucial in directing osteoblast differentiation^40–42^. The expression of *RUNX2* and *SP7* was significantly higher from day 7 in culture in osteogenic medium compared to undifferentiated cells. Addition of vitamin D increased the expression of *RUNX2* and *SP7* on day 7 but this was not significantly higher in comparison to osteogenic medium alone. Thus, the fundamental osteogenic mechanism of concomitant *RUNX2* and *SP7* expression, is reflected in our transdifferentiation process.

Our osteoblast-like cells also displayed significant upregulation of mRNA expression of other osteogenic markers such as *ALP*, *SPARC*, *OPN* and *COL1A1* from day 7 in culture. The expression of these markers was for all generally stable in the following days. We also measured *DMP1* expression, which is known to be expressed at the interface between late osteoblasts and early osteocytes. Our osteoblast-like cells showed gradually increased expression of *DMP1* from day 7 to day 21 in agreement with their gradual transition to more mature osteoblasts. Improper function of *DMP1* can delay osteoblast differentiation and downregulate the expression of osteoblast markers^43^. The treatment of the differentiated osteoblast-like cells with osteogenic media with platelet lysate is recommended for culture periods beyond 21 days during which the differentiated state of cells may potentially decrease as shown by the variable expression of *RUNX2* and *ALP* in the primary fibroblast cell cultures compared to day 21.

The RNA-seq data also supported the osteogenic properties of our osteoblast-like cells. Based on significantly upregulated genes in their gene expression profile, the osteoblast-like cells clustered together separately from the fibroblast cells indicating a distinct differential gene expression pattern as a result of the cell type switch. As expected, the expression of certain osteoblast markers such as *ALP* and *COL1A1* was higher in osteoblast-like cells. However, the expression of *SP7* was undetected in our RNA-seq data. This was an unexpected result because it was detected at mRNA level by qPCR. The possible explanation for this is the limitation of RNA-seq, owing to the vulnerability to general biases and errors caused by the variability in preferential sites of fragmentation, as well as variable primer and tag nucleotide composition effects^44,45^. Another possible reason may be the inability of the available method to detect certain transcripts and/or cover their entire length in combination with complicated gene structure, short transcript genes, overlapping gene models and gene family size^46,47^. Similarly, in a previous study which described the isolation and characterization of human primary osteoblasts from needle biopsy, RNA-seq also failed to detect *SP7* expression in contrast to qPCR analysis^48^.

Future analysis of the platelet lysate components will help to understand how they promote osteogenic pathways in the process of transdifferentiation^49^. We also found no significant increase in the expression of the chondrogenic markers *ACAN* and *SOX9* between transdifferentiated osteoblasts and primary fibroblasts and we did not detect any other chondrogenic marker expression such as *COL2A1*. The lack of differences was also confirmed by qPCR for *SOX9* and *ACAN*; this may suggest that the endochondral pathway is not reflected in our transdifferentiation model.

With its three dimensional (3D) and complicated structure, it is difficult to simulate bone structure in vitro^50,51^. An apparent limitation of our model is the two dimensional in vitro culture which may prohibit the terminal differentiation of osteoblasts to osteocytes; their critical role in sensing mechanical loading and conveying signals to regulate bone turnover is not reflected in our system^6^. Our further research aims the development of a 3D culture model in an effort to achieve osteocyte differentiation to faithfully reproduce bone tissue in vitro. A recent study has demonstrated the successful generation of a bone organoid by applying mechanical stimulation through fluid flow-derived shear stress on cells seeded on porous 3D silk fibroin scaffolds^52^. This may hint the requirement for mechanical stimulation in order to promote differentiation to the osteocyte level. However, forced expression of RUNX2 in combination with dexamethasone, forskolin and CHIR99021 treatment has been shown to generate osteocyte-like cells from human fibroblasts in the absence of a 3D environment^12^. Platelet-rich plasma (PRP) in combination with allogeneic MSCs has been used to treat osteoporotic bone defects in an ovariectomized rat model^53^. In addition, osteoblast-like cells generated from mouse embryonic fibroblasts by treatment with platelet-rich plasma, were able to improve bone quality in ovariectomized senescence-accelerated mice^54^. Future studies in in vivo models will show to which extent platelet lysate-treated fibroblasts could be in bone regeneration.

Fibroblasts owe their differentiation plasticity to their stem cell-like character which allows their direct differentiation to many other cell types by growth factor stimulation^55,56^. Human platelet lysate provides a cocktail of growth factors which favor the osteogenic differentiation of MSCs towards osteoblast-like cells^57^. Previously described protocols have reported the differentiation of osteoblasts from MSCs obtained from bone marrow by addition of platelet lysate but the invasiveness of bone marrow acquisition limits the availability of this technique especially in patients with increased chance of severe complications^52,57^. Platelet-derived products such as platelet gel, platelet rich plasma and platelet lysate have already been studied clinically and developed for animal free-derived product cellular therapies^58–60^. Platelet lysate is previously shown to be able to differentiate dental pulp stem cells to hepatic lineage cells, and MSCs to osteoblasts, adipocytes or vascular smooth muscle cells^61,62^. In the future, the transplantation of platelet lysate-based in vitro cultured products can be beneficial for cellular therapy in bone regeneration^63^. We have shown that our platelet lysate-based osteogenic media can be also used to induce osteogenic differentiation of human MSCs. Considering that MSCs and fibroblasts are considered by the International Society for Cellular Therapy to be the same cell type, it is tempting to speculate that a similar differentiation program is triggered in the two cell types by the platelet lysate-containing media^64^.

Our transdifferentiation method circumvents the need for genetic adjustments providing a reliable and time efficient way to produce patient-derived osteoblasts as a highly relevant disease model for the study of genetic bone diseases which can be potentially expanded to also address multifactorial bone disorders. The lack of genetic manipulation in combination with the immunocompatibility of patient-derived cells and platelet lysate also make this method attractive for bone tissue regeneration applications in patients requiring osteoblasts in their skeletal system. It also offers an alternative to the use of dexamethasone op which the ambiguous effects on bone regulation are not yet fully understood^65^. This method was used as a disease model for FOP in which increased potential for osteogenic transdifferentiation was observed in FOP patient fibroblasts in agreement with their propensity to develop heterotopic ossification; this study also allowed the identification of a novel disease target demonstrating its feasibility as a platform for therapeutic interventions^66^. Furthermore, our model clearly recapitulated the bone phenotype of the hypomyelination with spondylometaphyseal dysplasia (H-SMD) syndrome allowing the study of the mutated AIFM1 protein in the disease-relevant osteoblast-like cells. In conclusion, we provide an effective in vitro model to generate patient-specific osteoblast-like cells which can potentially aid the study of genetic bone disorders affecting osteoblast function and differentiation.

## Methods

### Human materials

Primary dermal fibroblasts were obtained from skin biopsies of 9 healthy controls. Permission was granted beforehand by the local medical ethics committee (METc VUmc) and all experiments were performed in accordance with local guidelines and regulations. Informed consent was obtained from all participants and/or their legal guardians. The sex of the healthy controls was variable male (n = 4) and female (n = 5) while the age was variable from 3 months to 57 years old. Human mesenchymal stem cells (Promocell) were from a 60-year-old donor. All primary fibroblast cultures used in this study tested negative for mycoplasma.

### Cell culture

Primary human dermal fibroblasts obtained from skin biopsies were cultured in Ham's F-10 Nutrient Mix medium (Gibco BRL) supplemented with heat-inactivated 10% fetal bovine serum (FBS, Gibco BRL) and 1% penicillin/streptomycin (Gibco BRL, Life Technologies, Ltd, Paisley, UK). When near confluence density was achieved, the cells were trypsinized and counted using a Coulter Counter® (Beckman Coulter, Indiana, USA). The osteogenic differentiation was performed in 12-well plates (Cellstar®, Greiner Bio-One GmbH, Frickenhausen, Germany) with a density of 100,000 cells per well after overnight cell attachment using the following conditions. 1. Fibroblast medium consisting of Ham's F-10 Nutrient Mix medium (Gibco BRL), 10% FBS (Gibco BRL) and 1% penicillin/streptomycin (Gibco BRL). 2. Osteogenic medium of minimal essential medium alpha (α-MEM) (Gibco BRL), 90 µg/ml L-ascorbic acid-2-phosphate (Sigma Aldrich Chemie NV, Zwijndrecht, The Netherlands), 5 mM β-glycerol-phosphate (Sigma Aldrich Chemie NV), 0.2% heparin (Leo Pharma, Ballerup, Denmark), 5% human platelet lysate (VU University blood bank) and 1% penicillin/streptomycin (Gibco BRL). 3. Osteogenic medium with 10 ng/ml vitamin D (Sigma Aldrich Chemie NV) for the first 3 days followed by osteogenic medium thereafter. Osteogenic medium was freshly made and replaced every 3–4 days during the osteogenic transdifferentiation timecourse. Primary human osteoblasts were cultured in minimum essential medium (α-MEM) also supplemented with heat-inactivated 10% FBS and 1% penicillin/streptomycin. Human mesenchymal stem cells were cultured in Mesenchymal stem cell growth medium 2 (Promocell). All cell cultures for this study were cultured in an environment of 37 °C and 5% CO_2_ in a humidified atmosphere.

### qPCR, RNA isolation and cDNA synthesis

RNA was isolated for timepoints day 0, 2, 3, 7, 14 and 21 with Quick-RNA MiniPrep (Zymo Research, Irvine, USA) according to the manufacturer’s instructions. The quality and quantity of RNA was measured with NanoDrop 1000 (Thermo Fisher Scientific Inc, Cleveland, USA). The cDNA was constructed with Reverse Transcriptase II (Thermo Fisher Scientific Inc) according to manufacturer’s protocol. Expression of osteoblast markers was measured with qPCR performed in 384-well plates using the LightCycler® 480 (Roche Diagnostics, Basel, Switzerland) with the following PCR program: pre-denaturation at 95 °C for 2 min followed by 45 cycles of denaturation at 95 °C for 10 s, annealing at 60 °C for 20 s and extension at 72 °C for 30 s. The primers (Invitrogen, Thermo Fisher Scientific, Cleveland, USA) were designed with the software LightScanner Primer Design (Idaho Technology Inc., Utah, USA) and are listed in Table 1.

**Table 1:** Sequence of the primers used for qPCR.

Gene name	Accession number	Primers	Product size (bp)
*RUNX2*	NM_001024630.3	ATGCTTCATTCGCCTCAC	156
		ACTGCTTGCAGCCTTAAAT	
*ALP*	NM_000478.5	AGGGACATTGACGTGATCAT	242
		CCTGGCTCGAAGAGACC	
*OPN*	NM_001040058.1	TTCCAAGTAAGTCCAACGAAAG	181
		GTGACCAGTTCATCAGATTCAT	
*SPARC*	NM_001309443	CTGTCCAGGTGGAAGTAGG	233
		GTGGCAGGAAGAGTCGAAG	
*DMP1*	NM_004407.3	TAGGCTAGCTGGTGGCTTCT	375
		AACTCGGAGCCGTCTCCAT	
*COL1A1*	NM_000088.3	GTGCTAAAGGTGCCAATGGT	128
		ACCAGGTTCACCGCTGTTAC	
*SP7*	NM_001173467.2	ACAAAGAAGCCGTACTCTG	146
		GGGTCATTAGCATAGCC	
*SOX9*	NM_000346	CCCAACGCCATCTTCAAGG	242
		CTGCTCAGCTCGCCGATGT	
*ACAN*	NM_001135.4	CAGTGCCTATCAGGACAAGGT	193
		AGAGATGGCTCTGTAATGGAAC	
*YWHAZ*	NM_145690.2	GATGAAGCCATTGCTGAACTTG	229
		CTATTTGTGGGACAGCATGGA	
*TBP*	NM_003194.4	AGTTCTGGGATTGTACCGCA	139
		TCCTCATGATTACCGCAGCA	

All samples were analyzed in duplicate using the LightCycler 480 Software release 1.5.0 SP4 (Roche Diagnostics). mRNA levels of the target genes were normalized to the housekeeping gene *YWHAZ* and *TBP* using the advanced relative quantification analysis. Gene expression analysis in chondrocytes was performed with Human Chondrocytes-articular Total RNA (Sanbio) after cDNA synthesis.

### Mineralization assay

Cells were washed with 2 ml EBSS (Gibco BRL) and fixed with 10% formaldehyde for 15 min at room temperature after 21 days in culture. For ARS staining, the cells were rinsed three times with MilliQ water followed by the incubation of cells with 1 ml/well Alizarin Staining Solution (EMD Millipore, Massachusetts, USA) at room temperature for 20 min. The cells were then washed with MilliQ water four times during five minutes on a shaking platform. Next 1 ml MilliQ water was added to each well to prevent drying of the cells.

ALP activity staining was performed after fixation with 10% formaldehyde by incubating the cells for 10 min in 0.1 M Tris-buffered saline with pH 7.6 followed by 10 min incubation in ALP buffer with pH 10–11. The cells were incubated with BCIP/NBT Color Development Substrate (Promega, Wisconsin, USA) solution for 5 min at 37 °C for ALP activity staining. To stop the activity, the cells were washed with deionized water.

Von Kossa staining was also carried out after fixation with formaldehyde; the cells were incubated with 1% silver nitrate solution with UV light for 30 min. After washing with EBSS, the cells were washed with 5% Natrium thiosulphate solution to remove unreacted silver. All stainings were visualized by scanning for macroscopic visualization while microscopic visualization was performed with the Leica DM IRB microscope. Mineralisation was quantified from the acquired images using Fiji^22^. Firstly, the images were converted into 8bit to make them monochrome prior to the quantification. Each well which was stained for mineralization was marked as a region of interest (ROI), and the mean gray value was quantified. Gray values range from 0 (black) to 255 (white), so the final numbers depicted in Fig. 1 were obtained by subtracting the measured value from 255.

### Immunostaining and quantification

After 21 days of osteogenic transdifferentiation, the cells were trypsinized and seeded on glass cover slips (Thermo Scientific). After 3 days, the cells were fixated with 10% formaldehyde solution for 15 min followed by washing three times in PBS (Gibco BRL) with 0.05% Tween (Sigma Aldrich Chemie) (PBST). The cells then were incubated in PBS with 0.2% Triton X-100 (Sigma Aldrich Chemie) for 15 min and afterwards incubated for 1 h in blocking buffer, consisting of PBS, 0.05% tween and 1% BSA (Sigma Aldrich Chemie). Subsequently, the cells were incubated overnight at 4 °C with primary antibodies for RUNX2 (Abcam, Cat No. AB23981 Cambridge, UK) with dilution 1: 100 and Osteocalcin with dilution 1:100 (Bioss, Cat No. bs-4917R, Massachusetts, USA). The following day, after washing with PBST at least 3 times, the cells were incubated at room temperature for 1 h with the Alexa Fluor 488 anti-rabbit and Alexa Flour 555 anti-rabbit secondary antibodies for RUNX2 and Osteocalcin, (1:1000;Thermo Fisher Scientific) DAPI (1:200; Thermo Fisher Scientific) and F-actin staining (phalloidin, 1:200; Thermo Fisher Scientific). The cover slips were mounted to slides, visualized and quantified with the Leica DM6000B microscope using the LASX software (Leica Microsystems, Wetzlar, Germany). Images were acquired using the Nikon RA1 (Nikon) microscope and the corresponding Nis-Elements C Software (Nikon). Representative images and images used for quantification of staining and cell morphology were analyzed and adjusted using Fiji^22^. RUNX2 and OCN staining intensity was quantified on the acquired images. For RUNX2, which is expressed in the nucleus, the ROI for quantification was marked based on the DAPI staining, and mean gray value was quantified. The acquired values were divided by the number of nuclei in the image to obtain the intensity of RUNX2 staining per cell. For OCN, the intensity of the staining was quantified in the whole cell by quantification of mean gray value. Again, the acquired values were divided by the number of nuclei in the image to obtain the intensity of OCN staining per cell.

Cell morphology was assessed by quantification of cell surface and shape. Cell surface was quantified by creating a region of interest using the phalloidin staining for F-actin, and subsequently marking the surface of the whole cell. The measured area values were divided by the number of nuclei in the image to obtain the average surface of individual cells.

Cell shape was assessed as a ratio between cell width and length. Cell width was measured using the *Straight* selection tool and manually drawing a line through the short axes in the middle of the spindle shaped cells, which was treated as the ROI. Cell length was assessed in the same way, by drawing a line orthogonally to the width and extending throughout the spindle shaped cell. The marked lines were measured using the Area function of Fiji measurements. Cell shape was then described as a ratio between cell width and length.

### RNA-seq

RNA libraries were prepared according to the manufacturer’s instructions for the Illumina TruSeq Stranded mRNA library prep kit (Illumina, Inc., California, USA). The libraries were sequenced with the HiSeq 2500 sequencing platform (Illumina, Inc.). The quantity and quality of RNA and library were measured respectively with RNA Analysis ScreenTape (Agilent Technologies, California, USA) and D1000 ScreenTape using 2200 TapeStation System (Agilent), respectively. The data was analyzed using several steps: For Read alignment and gene counts STAR 2.6.1d was used using the ENSEMBL hg38 reference build from Illumina iGenomes. The quality control is done using FastQC 0.11.4 and MultiQC v1.8 for summarization of the quality metrics. For the differential gene expression and normalization, the DESeq2 R package was used in combination with heatmap for the heatmaps. Significant differences between samples are expressed with False Discovery Rate (FDR) value less than 0.05. Further analysis with significantly different expression genes was performed with Metascape gene ontology (https://metascape.org/gp/index.html#/main/step1) to investigate biological gene functions and interactions.

### Western blotting analysis

Transdifferentiated primary fibroblasts and primary human osteoblasts were lysed in NuPAGE® LDS Sample Buffer with NuPAGE® reducing agent by scraping. The whole cell lysates were subjected to electrophoresis after which proteins were transferred to a nitrocellulose membrane with the iBLOT transfer system (Invitrogen). After incubation in blocking buffer for 1 h (LI-COR Biosciences), the nitrocellulose membrane was incubated with the primary antibodies against RUNX2 (abcam; Cat#ab23981) and actin (abcam; Cat#ab14128) overnight at 4 °C. Visualization of the signal was performed after incubation with the secondary antibodies IRDye 800 CW goat anti-rabbit IgG and the IRDye 680 CW goat anti-mouse IgG antibodies by using the Odyssey infrared imaging system equipped with the Odyssey version 4 software (LI-COR Biosciences). The relative RUNX2 expression is quantified using Image Studio Lite (LI-COR Biosciences).

### Statistical analysis

The Statistical Package for the Social Sciences (SPSS) version 22 (IBM, New York, USA) was used for statistical analysis. A repeated ANOVA measurement, corrected by a Tukey test, was performed to determine if there was a significant difference expression of the different bone specific markers in cells treated with fibroblast medium and osteoblast medium. The mean of those values was compared to 1 by a Wilcoxon signed-rank test. P values lower than 0.05 (*p* < 0.05) were considered to be significant. Graphs were created using GraphPad Prism7 (GraphPad software). Enrichment analysis between datasets was performed using a Fisher exact test in GraphPad Prism 8.

## Supplementary Information

Supplementary Information 1.

Supplementary Information 2.

## Data Availability

The RNAseq data which were generated and analysed during the current study are available in the GEO database repository, repo accession ID GSE206594.

## References

[1] Bonafe L (2015). Nosology and classification of genetic skeletal disorders: 2015 revision. Am. J. Med. Genet. Part A.

[2] Ralston SH (2007). Genetics of osteoporosis. Proc. Nutr. Soc..

[3] Masi L (2015). Taxonomy of rare genetic metabolic bone disorders. Osteoporos. Int..

[4] Boyce BF, Zuscik MJ, Zing L, Thakker RV, Whyte MP, Eisman JA, Igarashi T (2013). Biology of bone and cartilage. Genetics of Bone Biology and Skeletal Disease.

[5] Orimo H (2010). The mechanism of mineralization and the role of alkaline phosphatase in health and disease. J. Nippon Med. Sch..

[6] Schaffler MB, Kennedy OD (2012). Osteocyte signaling in bone. Curr. Osteoporos. Rep..

[7] Takahashi K, Yamanaka S (2006). Induction of pluripotent stem cells from mouse embryonic and adult fibroblast cultures by defined factors. Cell.

[8] Kato H (2015). Promoting effect of 1,25(OH)2 vitamin D3 in osteogenic differentiation from induced pluripotent stem cells to osteocyte-like cells. Open Biol..

[9] Phillips MD (2014). Directed differentiation of human induced pluripotent stem cells toward bone and cartilage: In vitro versus in vivo assays. Stem Cells Transl. Med..

[10] Jeon OH (2016). Human iPSC-derived osteoblasts and osteoclasts together promote bone regeneration in 3D biomaterials. Sci. Rep..

[11] Herberts CA, Kwa MSG, Hermsen HPH (2011). Risk factors in the development of stem cell therapy. J. Transl. Med..

[12] Li Y (2017). Direct conversion of human fibroblasts into osteoblasts and osteocytes with small molecules and a single factor, Runx2. bioRxiv.

[13] Lu ZF (2020). Reprogramming of human fibroblasts into osteoblasts by insulin-like growth factor-binding protein 7. Stem Cells Transl. Med..

[14] Cha H, Lee J, Park HH, Park JH (2020). Direct conversion of human fibroblasts into osteoblasts triggered by histone deacetylase inhibitor valproic acid. Appl. Sci..

[15] Ullah I, Subbarao RB, Rho GJ (2015). Human mesenchymal stem cells-current trends and future prospective. Biosci. Rep..

[16] Alonso-Goulart V (2017). Mesenchymal stem cells from human adipose tissue and bone repair: A literature review. Biotechnol. Res. Innov..

[17] Yeung KK (2017). Transdifferentiation of human dermal fibroblasts to smooth muscle-like cells to study the effect of MYH11 and ACTA2 mutations in aortic aneurysms. Hum. Mutat..

[18] Claeys L, Bravenboer N, Eekhoff EMW, Micha D (2020). Human fibroblasts as a model for the study of bone disorders. Front. Endocrinol. (Lausanne)..

[19] Soundararajan M, Kannan S (2018). Fibroblasts and mesenchymal stem cells: Two sides of the same coin?. J. Cell. Physiol..

[20] Golub EE, Boesze-Battaglia K (2007). The role of alkaline phosphatase in mineralization. Curr. Opin. Orthop..

[21] van de Peppel J, van Leeuwen JPTM (2014). Vitamin D and gene networks in human osteoblasts. Front. Physiol..

[22] Schindelin J (2012). Fiji: An open-source platform for biological-image analysis. Nat. Methods.

[23] N M (2015). Transduction of Oct6 or Oct9 gene concomitant with Myc family gene induced osteoblast-like phenotypic conversion in normal human fibroblasts. Biochem. Biophys. Res. Commun..

[24] Yamamoto K (2016). Generation of directly converted human osteoblasts that are free of exogenous gene and xenogenic protein. J. Cell. Biochem..

[25] Langenbach F, Handschel J (2013). Effects of dexamethasone, ascorbic acid and β-glycerophosphate on the osteogenic differentiation of stem cells in vitro. Stem Cell Res. Ther..

[26] Monterubbianesi R (2019). A comparative in vitro study of the osteogenic and adipogenic potential of human dental pulp stem cells, gingival fibroblasts and foreskin fibroblasts. Sci. Rep..

[27] Wang Y-H, Liu Y, Maye P, Rowe DW (2006). Examination of mineralized nodule formation in living osteoblastic cultures using fluorescent dyes. Biotechnol. Prog..

[28] Gregory CA (2004). An Alizarin red-based assay of mineralization by adherent cells in culture: Comparison with cetylpyridinium chloride extraction. Anal. Biochem..

[29] Javed A, Chen H, Ghori FY (2010). Genetic and transcriptional control of bone formation. Oral Maxillofac. Surg. Clin. North Am..

[30] Komori T (1997). Targeted disruption of Cbfa1 results in a complete lack of bone formation owing to maturational arrest of osteoblasts. Cell.

[31] Fujita T (2004). Runx2 induces osteoblast and chondrocyte differentiation and enhances their migration by coupling with PI3K-Akt signaling. J. Cell Biol..

[32] Wu H (2014). Genomic occupancy of Runx2 with global expression profiling identifies a novel dimension to control of osteoblastogenesis. Genome Biol..

[33] Long F (2012). Building strong bones: Molecular regulation of the osteoblast lineage. Nat. Rev. Mol. Cell Biol..

[34] Zaidi SK (2006). Alterations in intranuclear localization of Runx2 affect biological activity. J. Cell. Physiol..

[35] Amir LR (2007). Immunolocalization of sibling and RUNX2 proteins during vertical distraction osteogenesis in the human mandible. J. Histochem. Cytochem..

[36] Rathore B, Singh M, Kumar V, Misra A (2016). Osteocalcin: An emerging biomarker for bone turnover. Int. J. Res. Med. Sci..

[37] Gorski JP (2011). Biomineralization of bone: A fresh view of the roles of non-collagenous proteins. Front. Biosci. Landmark. Ed..

[38] Young MF (2003). Bone matrix proteins: Their function, regulation, and relationship to osteoporosis. Osteoporos. Int..

[39] Jang W-G (2012). BMP2 protein regulates osteocalcin expression via Runx2-mediated Atf6 gene transcription. J. Biol. Chem..

[40] Rashid H (2014). Sp7 and Runx2 molecular complex synergistically regulate expression of target genes. Connect. Tissue Res..

[41] Komori T (2010). Regulation of osteoblast differentiation by Runx2. Adv. Exp. Med. Biol..

[42] Zhang C (2010). Transcriptional regulation of bone formation by the osteoblast-specific transcription factor Osx. J. Orthop. Surg. Res..

[43] Sun Y (2015). Glycosylation of Dentin Matrix Protein 1 is critical for osteogenesis. Sci. Rep..

[44] Hansen KD, Brenner SE, Dudoit S (2010). Biases in Illumina transcriptome sequencing caused by random hexamer priming. Nucleic Acids Res..

[45] McIntyre LM (2011). RNA-seq: Technical variability and sampling. BMC Genom..

[46] Hirsch CD, Springer NM, Hirsch CN (2015). Genomic limitations to RNA sequencing expression profiling. Plant J..

[47] Ozsolak F, Milos PM (2011). RNA sequencing: Advances, challenges and opportunities. Nat. Rev. Genet..

[48] Fujita K (2014). Isolation and characterization of human osteoblasts from needle biopsies without in vitro culture. Osteoporos. Int..

[49] Miroshnychenko O, Chalkley RJ, Leib RD, Everts PA, Dragoo JL (2020). Proteomic analysis of platelet-rich and platelet-poor plasma. Regen. Ther..

[50] van der Plas A (1994). Characteristics and properties of osteocytes in culture. J. Bone Miner. Res..

[51] Boukhechba F (2009). Human primary osteocyte differentiation in a 3D culture system. J. Bone Miner. Res..

[52] A A (2020). An organoid for woven bone. Sci. Rep..

[53] Wei B (2016). Effect of mesenchymal stem cells and platelet-rich plasma on the bone healing of ovariectomized rats. Stem Cells Int..

[54] WC L (2009). Transplantation of embryonic fibroblasts treated with platelet-rich plasma induces osteogenesis in SAMP8 mice monitored by molecular imaging. J. Nucl. Med..

[55] Chang Y, Li H, Guo Z (2014). Mesenchymal stem cell-like properties in fibroblasts. Cell. Physiol. Biochem..

[56] Alt E (2011). Fibroblasts share mesenchymal phenotypes with stem cells, but lack their differentiation and colony-forming potential. Biol. Cell.

[57] Chevallier N (2010). Osteoblastic differentiation of human mesenchymal stem cells with platelet lysate. Biomaterials.

[58] Warnke PH (2013). A clinically-feasible protocol for using human platelet lysate and mesenchymal stem cells in regenerative therapies. J. Cranio-Maxillofacial Surg..

[59] de Leon JM (2011). The clinical relevance of treating chronic wounds with an enhanced near-physiological concentration of platelet-rich plasma gel. Adv. Skin Wound Care.

[60] Burnouf T (2013). Blood-derived biomaterials and platelet growth factors in regenerative medicine. Blood Rev..

[61] Vasanthan P (2014). Comparison of fetal bovine serum and human platelet lysate in cultivation and differentiation of dental pulp stem cells into hepatic lineage cells. Biochem. Eng. J..

[62] Ben Azouna N (2012). Phenotypical and functional characteristics of mesenchymal stem cells from bone marrow: Comparison of culture using different media supplemented with human platelet lysate or fetal bovine serum. Stem Cell Res. Ther..

[63] Altaie A, Owston H, Jones E (2016). Use of platelet lysate for bone regeneration -are we ready for clinical translation?. World J. Stem Cells.

[64] Ma H, Mp C, Cd B, F D (2009). Mesenchymal stem cells: The fibroblasts’ new clothes?. Haematologica.

[65] Zhou H, Cooper MS, Seibel MJ (2013). Endogenous glucocorticoids and bone. Bone Res..

[66] Micha D (2016). Inhibition of TGF?? signaling decreases osteogenic differentiation of fibrodysplasia ossificans progressiva fibroblasts in a novel in vitro model of the disease. Bone.

