# Supplementary material for: Osteogenic transdifferentiation of primary human fibroblasts to osteoblast-like cells with human platelet lysate
Source: Sci Rep. 2022 Aug 29;12:14686. doi: 10.1038/s41598-022-18512-1 (PMC9424276; doi:10.1038/s41598-022-18512-1)
Supplement: Supplementary file 1 — Supplementary Information 1. [file 41598_2022_18512_MOESM1_ESM.docx]

**Supplementary information**

**Osteogenic transdifferentiation of primary human fibroblasts to osteoblast-like cells with human platelet lysate**

Ferdy K Cayami, Lauria Claeys, Ruben de Ruiter, Bernard J Smilde, Lisanne Wisse, Natalija Bogunovic, Elise Riesebos, Lyra Eken, Irsan Kooi, Erik A Sistermans, Nathalie Bravenboer, Gerard Pals, Sultana MH Faradz, Daoud Sie, E. Marelise W. Eekhoff, Dimitra Micha

**Supplemental** **Figure 1**

**Comparison of Alizarin Red S (ARS), Alkaline phosphatase (ALP) and von Kossa stainings between transdifferentiated osteoblast-like cells and primary osteoblasts.** Human primary fibroblasts from 2 healthy donors (FIB1, FIB2) and osteoblasts from 1 healthy donor (PO) were treated with osteogenic media (OG) while fibroblasts and osteoblasts were maintained in the standard fibroblast (FIB) and primary osteoblast (PO) media respectively. On day 21 the indicated stainings were performed.

**Supplemental Figure 2**

**Quantification of RUNX2 and osteocalcin (OC) expression.** After 21 days of osteogenic transdifferentiation (OB) of 3 different healthy donor primary fibroblasts (FB) and primary osteoblasts (OB) (a). Representative immunofluorescence staining of RUNX2 and osteocalcin on lower magnification in a healthy donor (F3), after 21 days of osteogenic transdifferentiation (O3) and human primary osteoblasts (PO). RUNX2 and osteocalcin are shown in red, DAPI in blue and phalloidin in white (b).

**Supplemental** **Figure 3**

**Quantification of cell morphology.** Primary fibroblasts from healthy donors were transdifferentiated to osteoblast-like cells for 21 days. (a) Cell surface quantification (b) Cell width/cell length. F indicates the undifferentiated fibroblasts and O the transdifferentiated osteoblast-like cells on day 21 whereas PO indicates the primary human osteoblasts. Bars indicate the mean of cell surface and cell width/cell length divided by the number of nuclei respectively. Error bars show the standard error of measurements in 5 different cells’ fields per group.

**Supplemental Figure 4**

**The efficiency of osteogenic transdifferentiation was quantified by counting the RUNX2- and Osteocalcin (OC)-expressing cells after 21 days of incubation of primary fibroblasts from 6 healthy donors in osteogenic media.** The percentage was calculated based on the number of positive cells in a total count of minimal 200 cells per cell line. Bars indicate the mean of % per 6 cell lines and error bars the standard error.

**Supplemental** **Figure 5**

**Optimization of platelet lysate concentration for osteogenic transdifferentiation.** Primary fibroblasts of healthy donors were subjected to osteogenic transdifferentiation with different concentrations of platelet lysate (0.5%, 1.0%, 2.0% and 5%) and the standard media for fibroblasts with FBS. The relative expression of *RUNX2* (a) and *ALP* (b) was measured on day 7, 14 and 21 after normalization to the expression of *YWHAZ*. Values are expressed as mean ± SEM per group of 4 cell lines.

**Supplemental Figure 6**

**Expression of osteogenic markers** *RUNX2*, *ALP*, *SPARC*, *OPN*, *SP7*, *COL1A1* and *DMP1* in primary human osteoblasts as compared to primary fibroblasts, transdifferentiated osteoblast-like cells and transdifferentiated osteoblast-like cells with vitamin D on day 21 (day 21 conditions are copied from Figure 4). Gene expression was normalized to *YHWAZ*.

**Supplemental Figure 7**

**MSCs were treated with the platelet-lysate based osteogenic media.** At the indicated timepoints, the expression of *RUNX2* and *ALP* was analyzed with qPCR. *TBP* was used to normalize relative gene expression. MSC indicates mesenchymal stem cells; OB indicates osteoblasts. n=2

**Supplemental Figure 8**

**RNAseq analysis was performed with 2 healthy control primary fibroblasts, before (FIB) and after 21 days of osteogenic transdifferentiation (OG).** The analysis also included 2 primary human osteoblast cell lines (PO). The figure shows an overview of the significantly upregulated genes, 198 genes are significantly upregulated in both primary osteoblasts and transdifferentiated osteoblast-like cells compared to primary fibroblast cultures.

**Supplemental Figure 9**

**Expression of chondrogenic markers at the first week of osteogenic transdifferentiation.** Primary fibroblasts from 4 healthy donors we subjected to osteogenic transdifferentiation (OG) for 1 week during which the expression of *SOX9* and *ACAN* was tested by qPCR at the indicated timepoints (day 1, 2, 3 and 7). Expression of these was normalized based on the expression of *TBP*. Bars indicate the mean expression per 4 cell lines per condition end error bars ±standard error. The expression of *SOX9* and *ACAN* was found to be 3 and 35.674 in primary human chondrocytes and 0.18 and 2531.4 in primary human osteoblasts respectively.

**Supplemental Figure 10**

**The full-length blot of Figure 3.** RUNX2 expression determined by western blotting analysis of primary fibroblasts from 5 healthy controls (F1-F5) and their transdifferentiated osteoblast-like cells on day 21 of osteogenic transdifferentiation (O1-O5) and primary osteoblasts (PO). Actin was used a loading control.

**Supplemental Table 1**

**List of significantly upregulated genes measured by RNA sequencing.**

Significant differences between samples are expressed with P-value less than 0.05. OB; osteoblast-like cells, FB; primary fibroblasts, PO; primary osteoblasts.
