# Supplementary material for: Osteogenic transdifferentiation of primary human fibroblasts to osteoblast-like cells with human platelet lysate
Source: Sci Rep. 2022 Aug 29;12:14686. doi: 10.1038/s41598-022-18512-1 (PMC9424276; doi:10.1038/s41598-022-18512-1)
Supplement: Supplementary file 2 — Supplementary Information 2. [file 41598_2022_18512_MOESM2_ESM.pdf]

Supplemental figures

Osteogenic transdifferentiation of primary human fibroblasts to osteoblast-like cells with human platelet lysate

Ferdy K Cayami[1,2*], Lauria Claeys[1*], Ruben de Ruiter[3], Bernard J Smilde[3], Lisanne Wisse[1], Natalija Bogunovic[1], Elise Riesebos[1], Lyra Eken[1], Irsan Kooi[1], Erik A Sistermans[1], Nathalie Bravenboer[4], Gerard Pals[1], Sultana MH Faradz[2], Daoud Sie[1], E. Marelise W. Eekhoff[3], Dimitra Micha[1]

Supplemental Figure 1

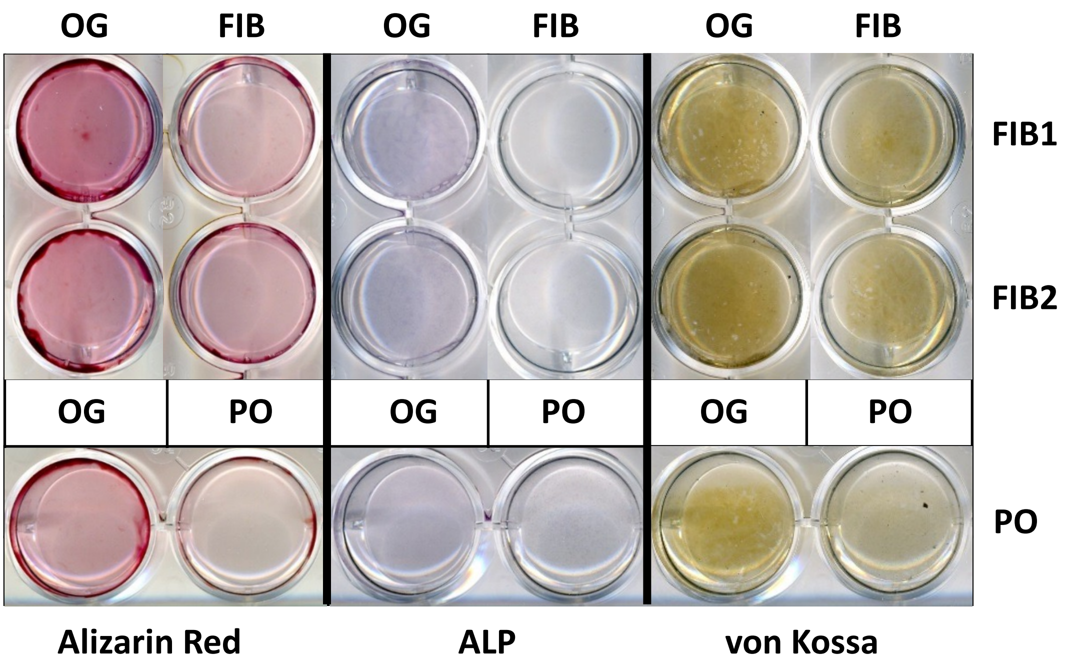

Supplemental Figure 2

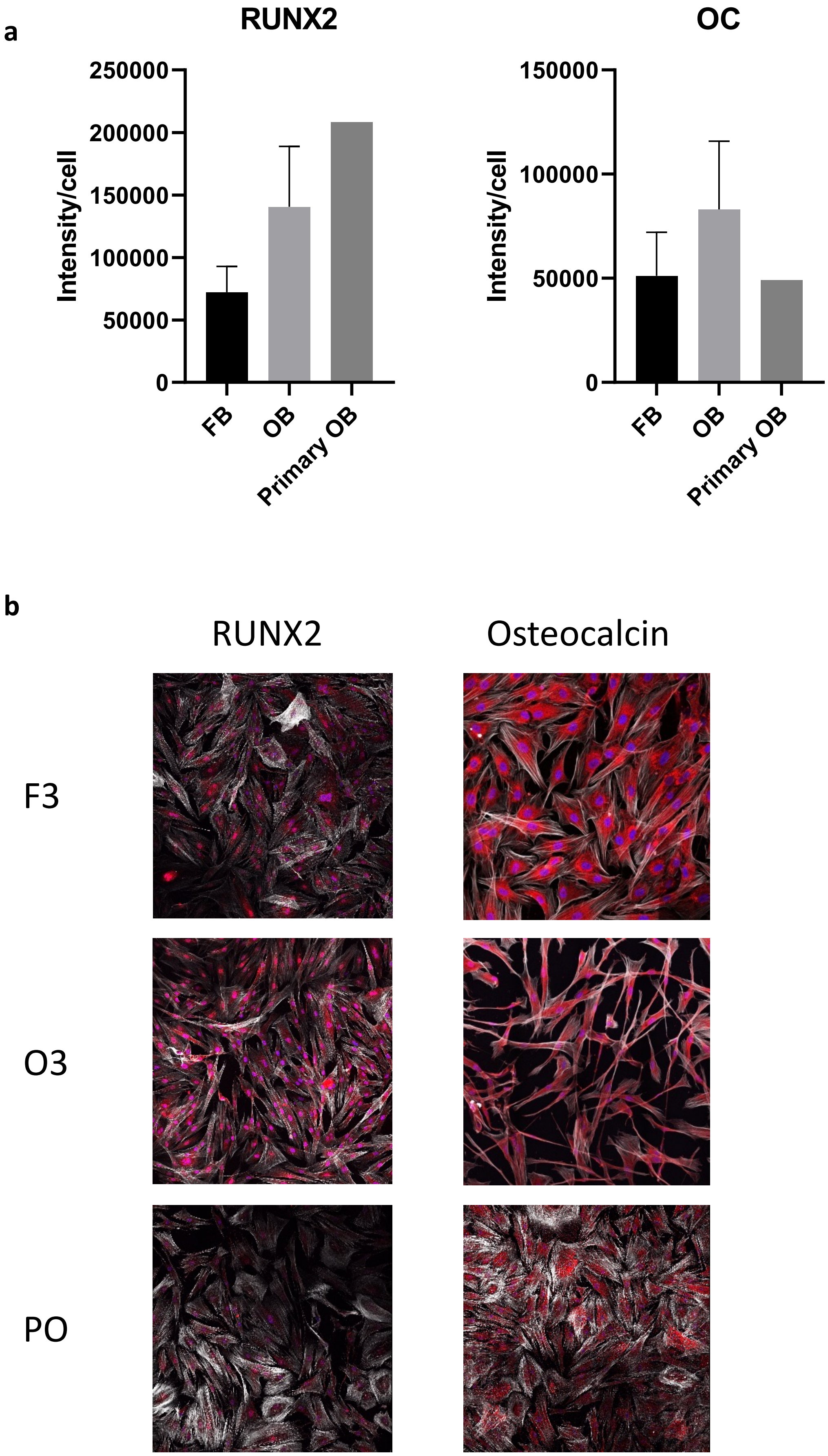

Supplemental Figure 3

a

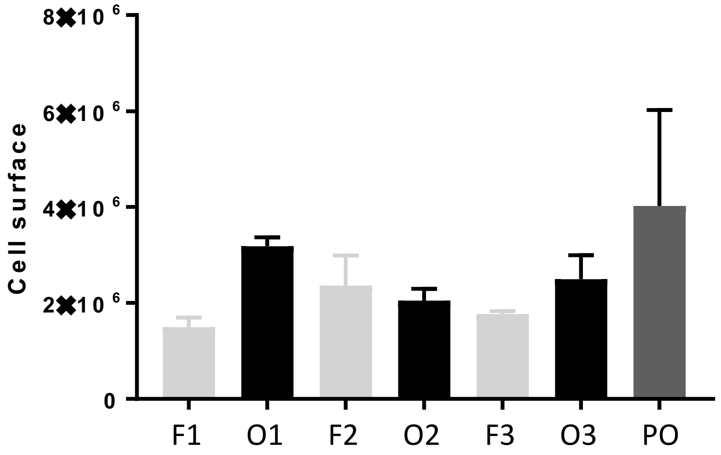

b

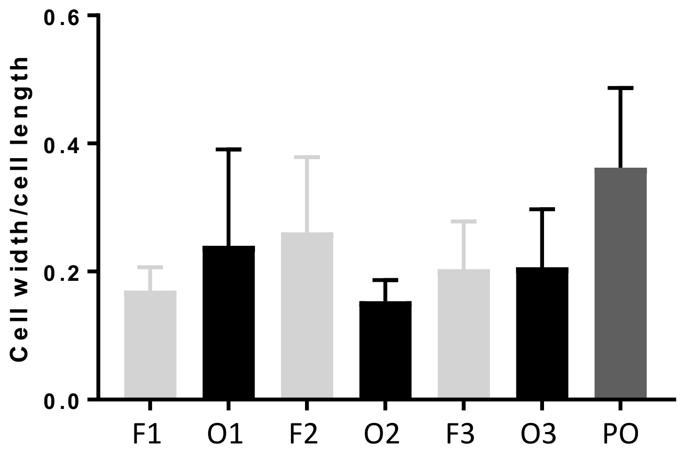

Supplemental Figure 4

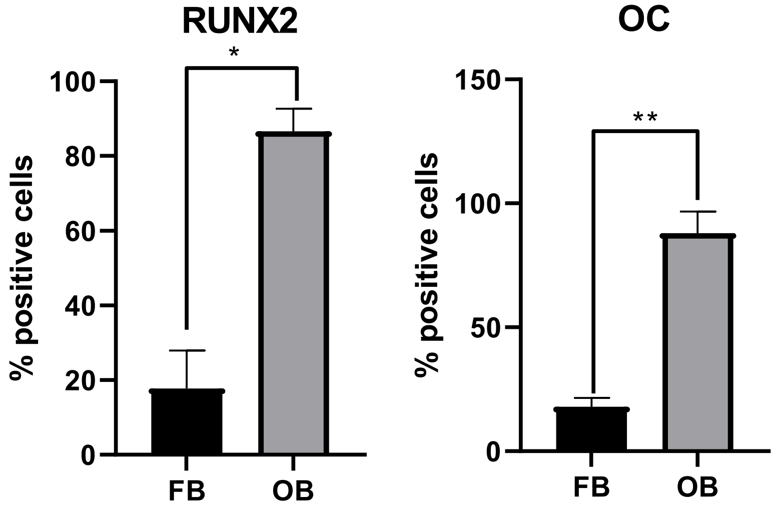

Supplemental Figure 5

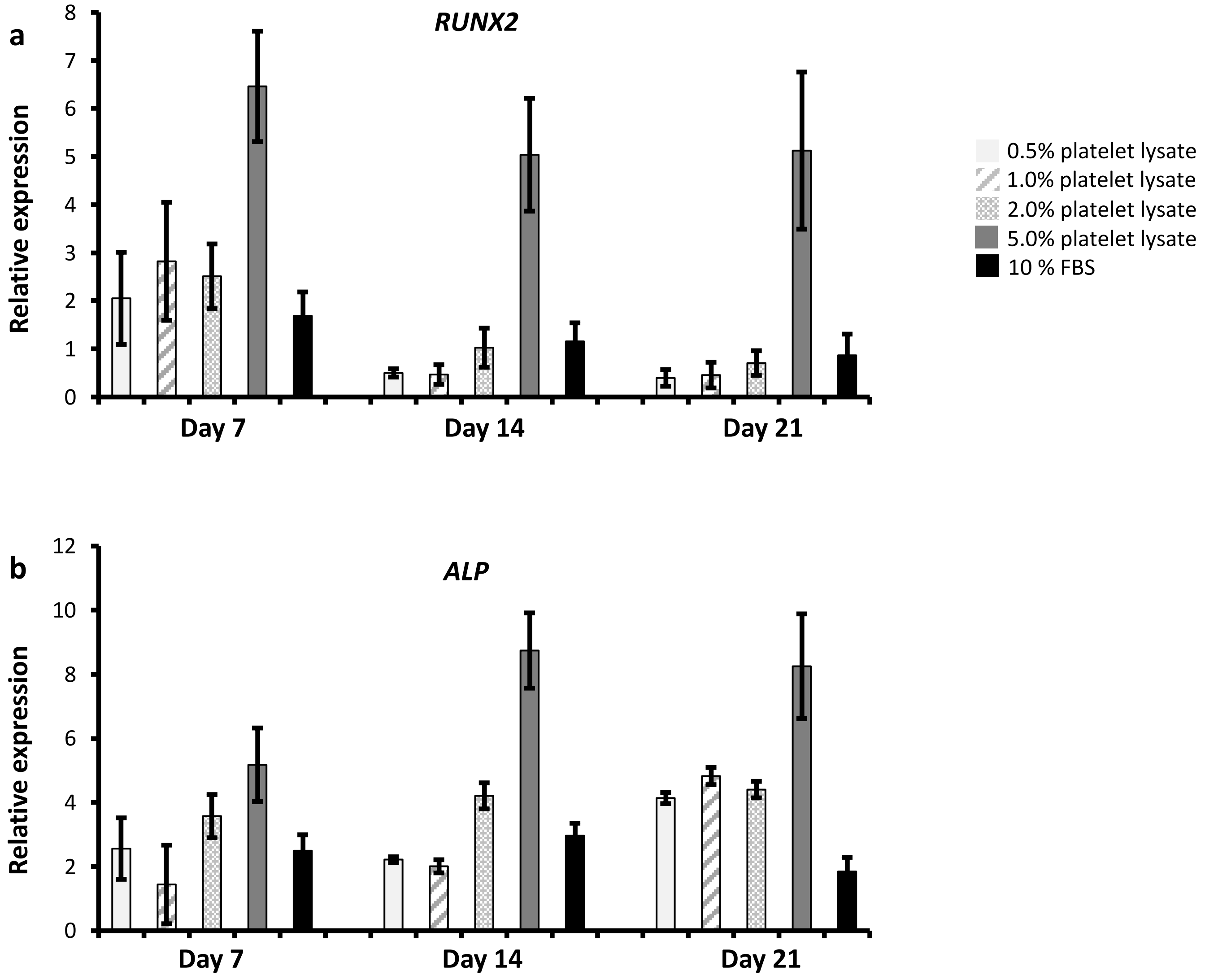

Supplemental Figure 6

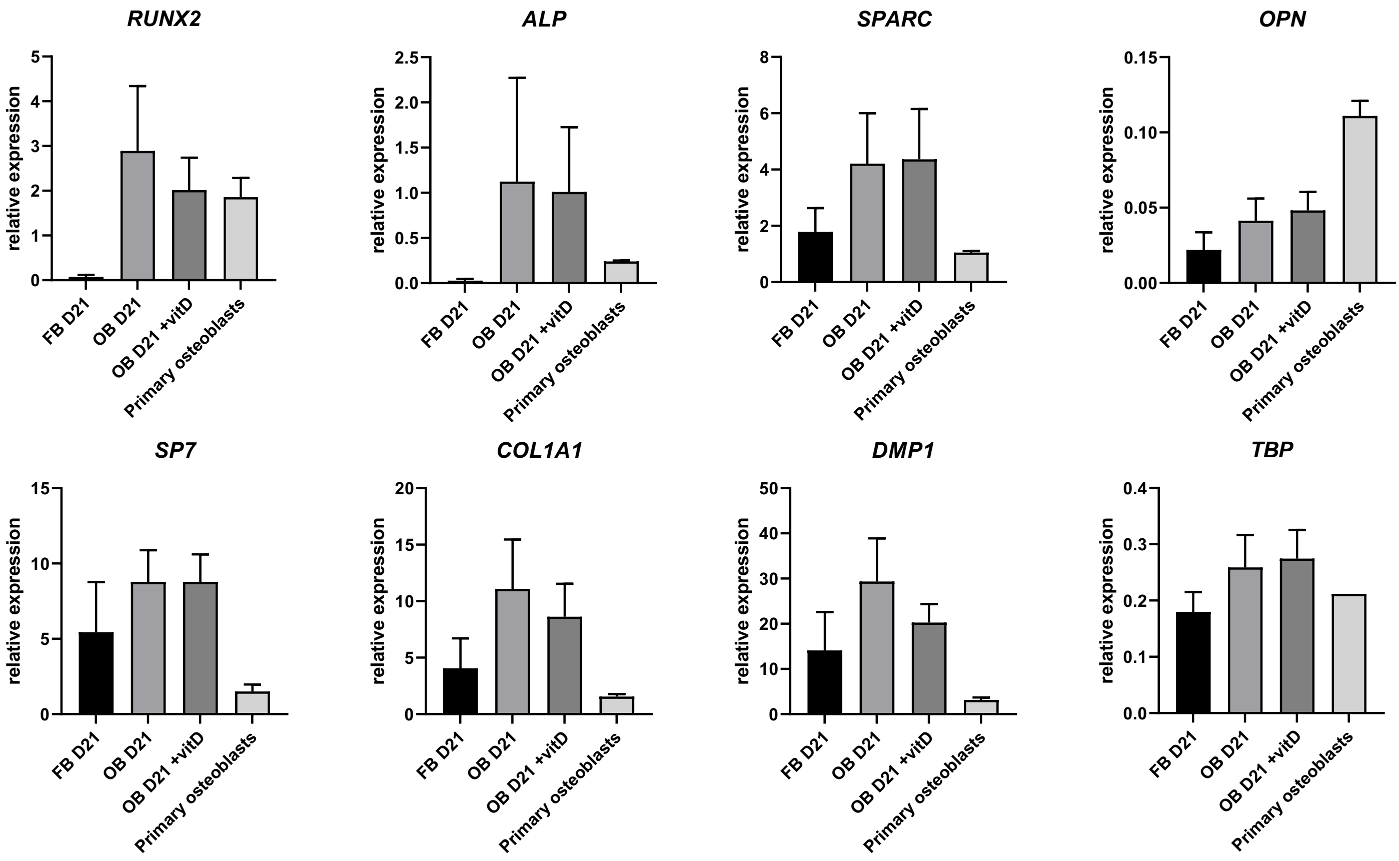

Supplemental Figure 7

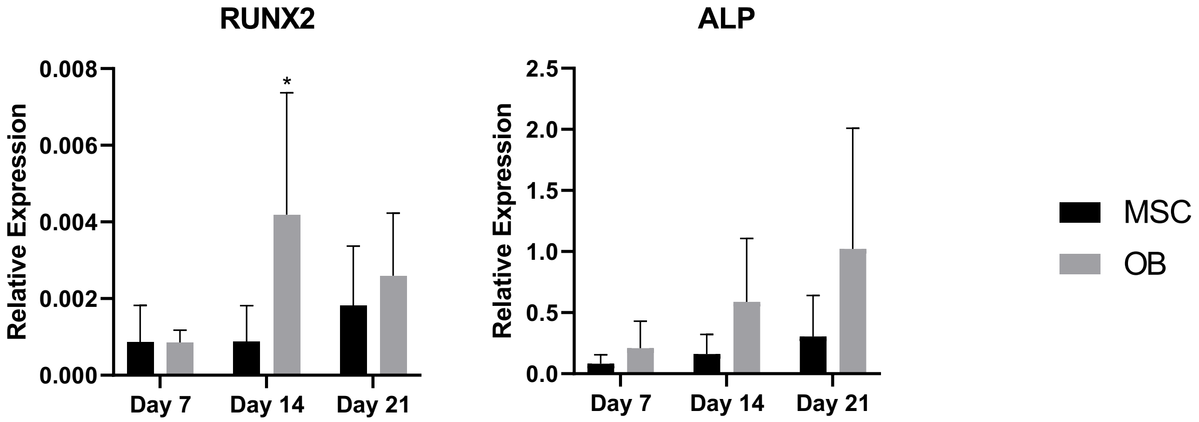

Supplemental Figure 8

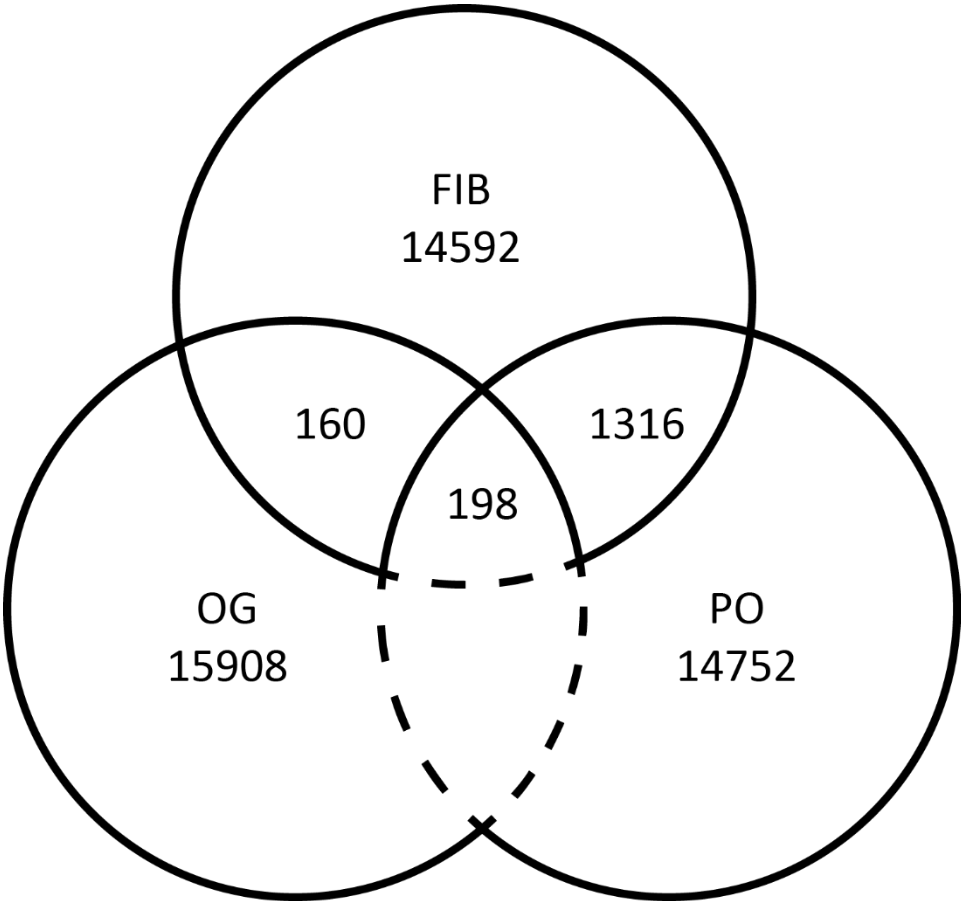

Supplemental Figure 9

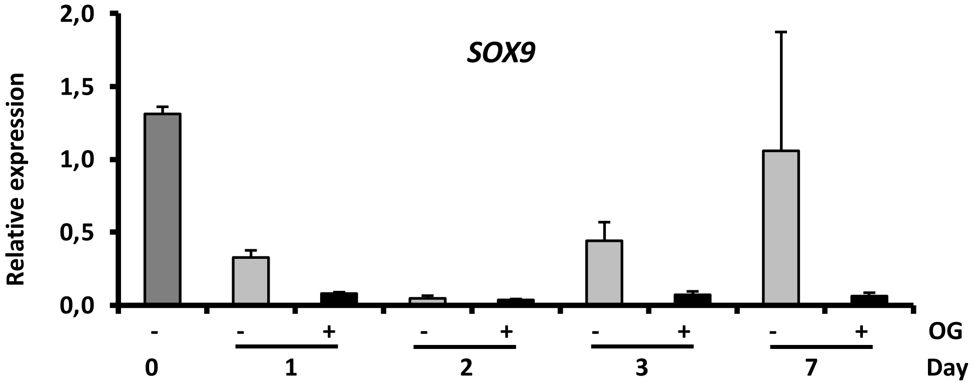

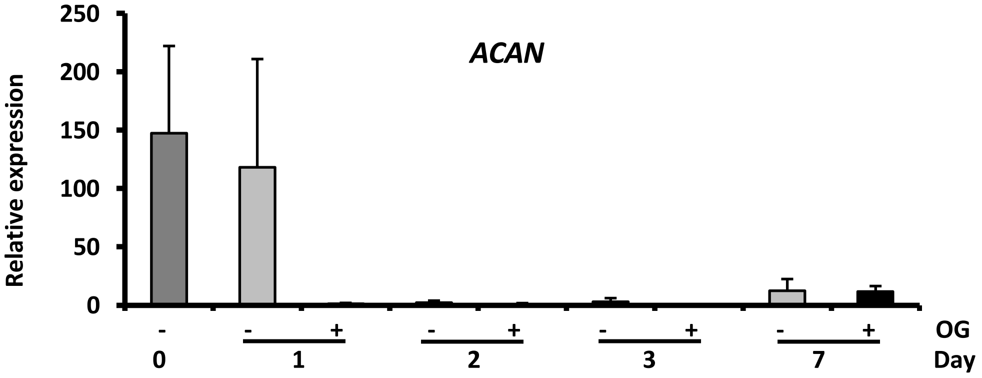

Supplemental Figure 10

a

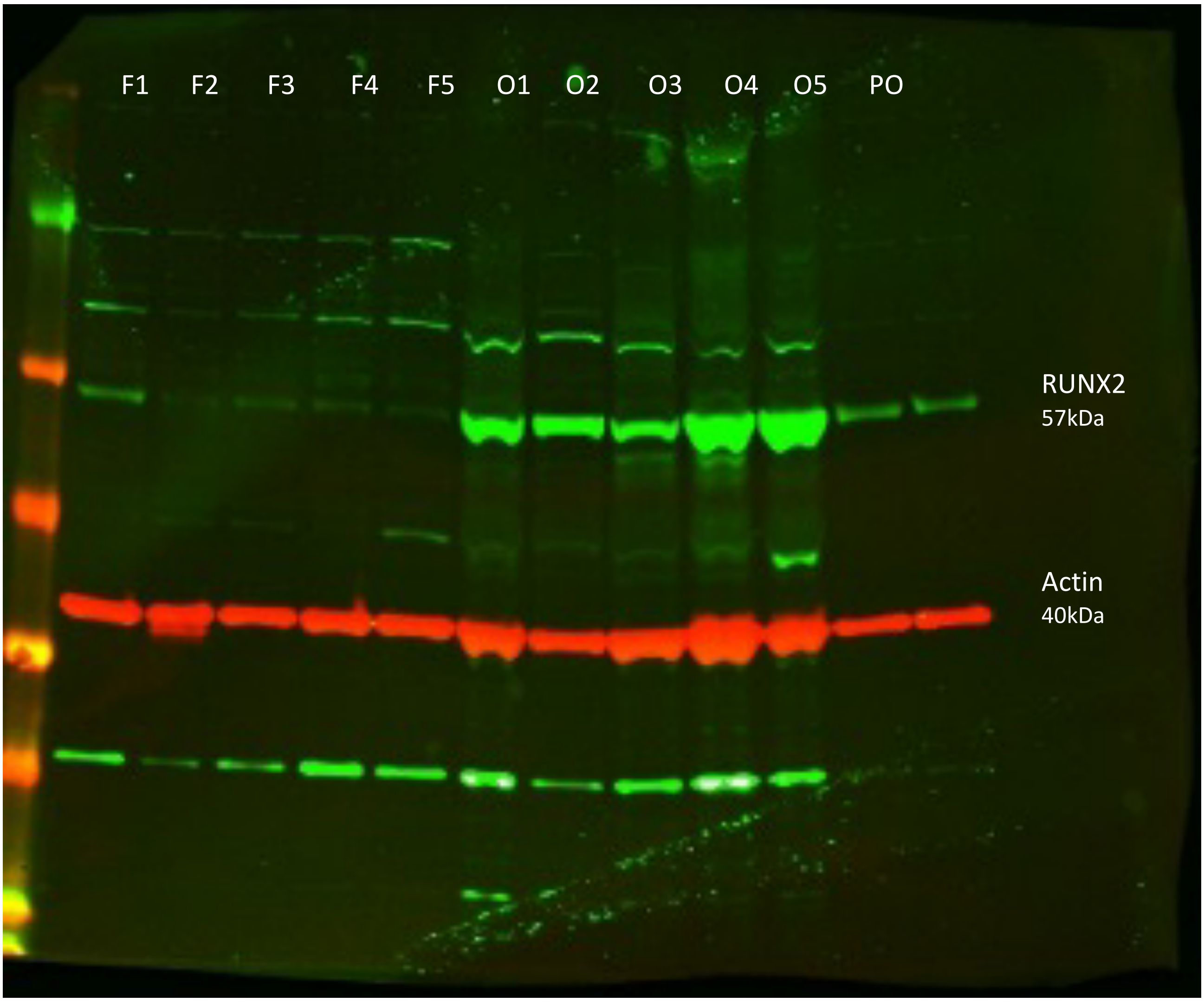

Supplemental Table 1

List of significantly upregulated genes measured by RNA sequencing.

Genes	FC OB/FB	P-value OB/FB	FC PO/FB	P-value PO/FB
STEAP4	21,90	7,66E-21	185,08	2,12412E-65
CHI3L1	6,40	6,79E-15	117,09	2,3064E-109
SLC43A3	7,76	2,14E-14	5,56	6,98344E-11
HSD17B14	21,23	1,08E-11	20,43	1,5615E-12
ITGA10	5,80	6,65E-10	61,17	5,75925E-68
AEBP1	2,74	1,25E-09	6,90	7,09134E-40
PTGES	3,64	3,62E-09	2,46	3,06282E-05
LAMA1	10,60	3,62E-09	40,89	7,33862E-26
CHST2	6,71	3,90E-09	5,22	9,44977E-08
DPP4	3,05	4,48E-09	1,65	0,016145271
SNX8	3,85	8,64E-09	3,06	7,81107E-07
CNIH3	6,97	8,64E-09	7,58	1,0431E-10
LRRC32	3,73	5,78E-08	3,39	7,84668E-08
MMP14	2,45	5,90E-08	3,63	2,14727E-18
SLC7A8	3,33	7,17E-08	26,80	3,91639E-71
CYSTM1	3,32	2,01E-07	4,08	1,73555E-11
FADS2	2,73	2,62E-07	15,90	8,05839E-65
RASD1	9,63	3,15E-07	15,13	7,89647E-12
GPC4	4,54	5,19E-07	42,74	1,72789E-54
PLTP	3,61	5,81E-07	1,81	0,034063507
EVI2A	50,75	7,53E-07	459,68	4,57493E-19
OLFML2A	3,99	9,73E-07	3,26	9,5392E-06
CTSK	4,86	1,21E-06	6,83	5,47367E-11
AMDHD2	3,52	1,51E-06	4,55	1,03229E-10
ECM1	3,98	1,76E-06	2,92	0,000116484
GLMP	3,08	1,92E-06	4,59	3,12344E-13
INSIG1	3,58	1,92E-06	15,44	1,41107E-33
UAP1L1	3,62	1,93E-06	4,36	1,60459E-09
RPLP0P2	12,30	2,33E-06	8,19	3,96024E-05
STX3	5,03	3,30E-06	4,46	4,033E-06
FABP3	2,96	3,76E-06	26,07	4,09071E-67
BCL2L1	2,60	3,95E-06	2,64	3,14573E-07
PEPD	2,60	4,70E-06	2,64	3,76214E-07
ETV1	3,49	4,82E-06	4,00	1,96759E-08
GBA	3,47	6,56E-06	5,26	7,64406E-12
PRXL2C	3,29	6,91E-06	2,18	0,003714947
HDAC9	5,77	7,22E-06	10,76	2,49361E-12
MTSS1	3,65	1,08E-05	37,95	5,10726E-55
BRI3	2,51	1,23E-05	2,97	6,71712E-09
RUBCNL	4,91	4,58E-05	11,31	8,44757E-14

NAGLU	2,66	5,99E-05	3,84	1,11896E-10
MX1	9,58	6,14E-05	6,02	0,000795632
RGS3	3,42	6,38E-05	10,52	2,45227E-20
FAM180A	2,67	7,28E-05	7,93	1,91914E-25
PLXDC1	12,22	7,28E-05	3,90	0,038818527
TMEM38B	4,17	7,92E-05	3,20	0,000756843
OAS2	16,22	9,00E-05	18,13	5,10736E-06
SLC17A5	2,33	0,000104089	3,33	8,80029E-11
FRMD5	11,78	0,000116693	24,83	3,42049E-09
LHFPL2	2,25	0,000167453	4,57	5,16866E-18
IRAK1	2,38	0,000208357	4,72	7,18561E-16
EMP1	2,29	0,000234596	2,63	8,29859E-07
NEU1	3,02	0,000234596	3,39	3,46957E-06
OAS1	20,69	0,000234606	10,27	0,003033623
PIP4K2A	2,74	0,000261372	1,96	0,014003653
ADGRL4	4,00	0,000305854	4,41	1,16604E-05
TCN2	3,47	0,000305854	4,51	3,80229E-07
BHLHE40	3,33	0,000346368	11,08	4,03386E-19
CTNS	3,44	0,000546515	2,72	0,002822182
ATP6V0D1	2,17	0,000588881	2,48	2,55E-06
NPC2	3,18	0,000688876	5,32	4,2196E-09
CCN4	3,85	0,000731248	90,84	1,14836E-55
PDE7B	5,85	0,000844464	3,85	0,007117576
NRROS	20,52	0,000881453	32,05	8,00734E-06
SCG2	11,53	0,00096365	111,52	1,30246E-16
STARD4	2,24	0,001010191	1,75	0,018692464
STON2	12,27	0,001218824	134,35	1,89778E-16
MCOLN3	18,51	0,001246368	40,88	7,58579E-07
FOLR3	9,24	0,001294203	5,75	0,007574319
SMIM29	3,54	0,001294203	3,26	0,000809574
KYNU	272,46	0,001375983	162,89	0,001324135
DUSP3	2,41	0,001405472	3,32	1,61262E-07
HABP4	2,27	0,001478341	3,88	6,65073E-11
FADS1	2,48	0,001478341	9,85	4,4097E-25
ABRACL	3,70	0,001487556	5,83	2,48922E-07
HEXB	2,64	0,001549193	2,17	0,00633508
MRPL41	2,48	0,001587311	1,86	0,028432175
SDCBP	1,95	0,001587311	1,85	0,001219783
ACTB	2,23	0,001587311	3,59	6,52658E-10
OGDH	2,02	0,001623516	2,10	0,000146274
RETREG1	15,83	0,001761861	19,56	0,000100095
LAMTOR1	1,99	0,001761861	1,73	0,007791951
RAB3IL1	2,14	0,001830125	1,77	0,013629932
ATP1B3	2,77	0,002012834	2,29	0,006453787
TPP1	2,43	0,002042296	6,04	2,30988E-15

PLEKHO1	2,63	0,002043021	3,11	2,09928E-05
LRRC59	1,87	0,002058452	2,11	1,52324E-05
P4HA3	5,30	0,002384279	16,50	1,26326E-10
CSTB	1,98	0,002392646	2,56	3,89138E-07
ABI3BP	2,69	0,002393253	11,39	6,11197E-22
HS1BP3	2,38	0,002446815	2,64	7,98981E-05
ARMC9	2,69	0,002486198	4,81	2,56945E-09
CDCP1	9,32	0,002541647	6,82	0,004335859
GOLGA4	1,81	0,003197887	1,87	0,000317983
LDLR	2,81	0,003246388	8,93	7,20056E-16
COL10A1	17,45	0,003304519	116,05	2,59238E-10
NAPA	2,17	0,003326593	3,08	1,29821E-07
ITPKB	2,49	0,003634888	2,50	0,000861418
KIAA1217	2,38	0,003695605	12,48	4,6495E-30
GNPDA1	2,39	0,00378681	3,15	3,53966E-06
SPP1	24,39	0,003816423	435,52	4,92675E-13
LIMK1	1,91	0,004070144	1,77	0,004873948
PHLDA1	3,84	0,004189	3,60	0,001968408
RAB7A	1,76	0,004254053	1,85	0,000307817
TIMP4	8,49	0,004317792	4,47	0,042078857
NEDD9	3,44	0,004669981	6,96	1,61601E-08
TRPV2	3,87	0,005056705	29,33	7,72353E-23
SPHK1	4,18	0,005085751	4,64	0,000437281
VEGFB	1,87	0,005208112	3,10	1,30062E-10
IL4I1	33,27	0,005236634	82,95	1,63984E-05
AC097359,2	7,12	0,0053247	7,51	0,000930717
GPNMB	3,28	0,005435085	5,91	2,2631E-07
SNTA1	2,40	0,005442205	1,98	0,019291537
KCNK15	2,92	0,005651518	6,01	2,94297E-09
TMEM192	2,54	0,005752336	3,78	8,85534E-07
ARHGDIA	2,09	0,006259777	2,76	3,69759E-06
CRYM	49,24	0,00662519	156,57	1,25707E-05
C11orf96	5,73	0,006650575	7,66	0,000146274
RFLNB	2,41	0,007074216	1,88	0,041494619
SOD2	3,44	0,007094024	2,41	0,041374546
ATP6V1B2	1,88	0,007142169	3,18	1,87016E-10
EHD4	2,36	0,007234073	6,91	5,07245E-16
H2AJ	2,11	0,007303129	1,74	0,033232346
ITGB5	1,65	0,007981322	4,63	1,30971E-28
TPM4	1,66	0,008379902	2,40	4,61038E-09
FGF5	2,01	0,008770494	1,97	0,003277656
DPP7	1,95	0,009127509	3,13	1,04039E-08
AL355607,2	15,28	0,009153477	10,33	0,013854639
ARHGAP24	2,24	0,009315174	3,21	1,90881E-06
SCD	3,02	0,009510423	49,85	4,81793E-37

PLXDC2	5,23	0,009540641	442,17	9,4714E-46
ENPP1	2,99	0,009822851	5,27	6,07527E-07
ETV5	2,16	0,009822851	2,09	0,004621587
PAG1	8,02	0,009822851	5,00	0,03051529
PCYT2	2,26	0,010059158	4,08	6,77125E-09
HSD3B7	2,15	0,010353121	1,97	0,010432064
TM4SF1	3,71	0,010489866	14,47	5,26073E-12
MCTP1	13,52	0,010915534	39,41	3,67888E-06
SQSTM1	2,00	0,011169116	2,31	0,000208695
PGAP6	2,19	0,011205131	3,60	8,34795E-08
RPP25	5,11	0,011226283	4,95	0,004109463
GUK1	1,83	0,011717152	1,86	0,002428736
WIPI1	2,53	0,012273042	2,18	0,018473957
MSMO1	1,79	0,01273187	7,09	7,37986E-33
EVI2B	23,79	0,012790984	101,70	2,38967E-06
SOAT1	1,91	0,012790984	2,06	0,000809574
CFL1	1,98	0,013128411	2,67	6,56184E-06
PCSK1	10,10	0,013289353	10,68	0,002837172
PSEN2	2,37	0,013360904	2,47	0,002179224
CTSD	2,01	0,014666918	6,21	2,10476E-18
C15orf48	92,52	0,01527695	34,65	0,038525722
CPED1	2,05	0,015502551	1,84	0,019954864
CCDC86	2,43	0,015573818	2,09	0,025769891
PDPN	4,25	0,0159015	4,50	0,002996338
HMGA2-AS1	6,88	0,01712271	7,53	0,003005685
FLCN	2,26	0,019055066	1,96	0,029879085
FOXM1	2,48	0,019367676	2,20	0,021534725
NMB	2,70	0,019438941	10,05	5,65626E-15
ENTPD6	1,99	0,021225204	2,26	0,000751189
ABL2	2,44	0,021866151	3,62	1,89544E-05
OGFRL1	2,27	0,022042689	2,03	0,023152999
CLCN7	2,52	0,022142461	3,32	0,000189824
FRZB	16,50	0,022559794	27,26	0,000828046
TBC1D2	2,57	0,023764168	8,64	7,76579E-13
ACTG1	2,14	0,023966324	3,71	2,50368E-07
GRN	1,85	0,024239684	3,47	6,27506E-10
MGLL	2,46	0,025102937	2,95	0,000782411
LRRFIP1	1,97	0,026261563	2,47	0,000141372
LAMP1	1,63	0,026730959	3,00	3,94155E-12
SH2D5	9,44	0,027433565	10,06	0,006278732
KIF13B	2,93	0,027574135	6,76	6,8658E-08
CD276	2,23	0,027574135	3,55	3,91236E-06
TMSB4X	2,68	0,0277536	2,26	0,037705441
MTHFD1	1,89	0,028336307	2,88	1,1646E-06
HSPA8	1,69	0,028968108	1,71	0,007379901

MYO1D	3,09	0,030175842	30,75	4,06348E-21
EEPD1	2,68	0,030553577	4,85	2,46226E-06
HEXA	2,21	0,030810861	3,62	2,95795E-06
LSS	2,22	0,03167219	2,79	0,000432935
GRINA	1,88	0,03179588	2,13	0,001287678
AC124798,1	5,74	0,032106122	10,25	0,00020349
NR4A3	8,10	0,03257847	8,00	0,011060793
MAP2K3	2,23	0,03257847	3,58	5,68865E-06
VAC14	2,04	0,034196212	3,26	2,34316E-06
HPS1	1,73	0,034664601	2,13	0,000154778
MARCHF2	2,00	0,034703848	3,61	6,47455E-08
THEMIS2	4,52	0,03628034	17,22	1,72959E-08
DUSP23	2,81	0,036457644	3,36	0,002090243
TNC	3,24	0,037372897	5,23	0,000136072
CPE	2,29	0,037433725	4,60	1,04733E-07
STK10	2,48	0,043742347	2,55	0,010938268
EPAS1	2,26	0,045559679	3,49	4,07083E-05
ABR	1,82	0,047554351	4,31	3,2001E-12
BNC1	3,73	0,04815063	5,36	0,001051916
RRAGC	1,83	0,048542044	2,50	6,26721E-05
P2RX4	2,39	0,048542044	4,55	2,06742E-06
AFAP1L1	3,73	0,049365871	5,32	0,0012006
RTN4RL2	6,02	0,049976543	5,79	0,021121457

Significant differences between samples are expressed with P-value less than 0.05. OB; osteoblast-like cells, FB; fibroblasts, PO; primary osteoblasts.
